# Structural and biochemical characterisation of Co^2+^-binding sites on serum albumins and their interplay with fatty acids†

**DOI:** 10.1039/d3sc01723k

**Published:** 2023-05-23

**Authors:** Dongmei Wu, Michal Gucwa, Mateusz P. Czub, David R. Cooper, Ivan G. Shabalin, Remi Fritzen, Swati Arya, Ulrich Schwarz-Linek, Claudia A. Blindauer, Wladek Minor, Alan J. Stewart

**Affiliations:** a School of Medicine, University of St Andrews St Andrews UK ajs21@st-andrews.ac.uk +44 (0)1334 463546; b Department of Molecular Physiology and Biological Physics, University of Virginia School of Medicine Charlottesville VA 22908-0736 USA wladek@iwonka.med.virginia.edu +1 434-243-6865; c Doctoral School of Exact and Natural Sciences, Jagiellonian University Krakow Poland; d Biomedical Sciences Research Complex, University of St Andrews St Andrews UK; e Department of Chemistry, University of Warwick Coventry UK

## Abstract

Serum albumin–Co^2+^ interactions are of clinical importance. They play a role in mediating the physiological effects associated with cobalt toxicity and are central to the albumin cobalt binding (ACB) assay for diagnosis of myocardial ischemia. To further understand these processes, a deeper understanding of albumin–Co^2+^ interactions is required. Here, we present the first crystallographic structures of human serum albumin (HSA; three structures) and equine serum albumin (ESA; one structure) in complex with Co^2+^. Amongst a total of sixteen sites bearing a cobalt ion across the structures, two locations were prominent, and they relate to metal-binding sites A and B. Site-directed mutagenesis and isothermal titration calorimetry (ITC) were employed to characterise sites on HSA. The results indicate that His9 and His67 contribute to the primary (putatively corresponding to site B) and secondary Co^2+^-binding sites (site A), respectively. The presence of additional multiple weak-affinity Co^2+^ binding sites on HSA was also supported by ITC studies. Furthermore, addition of 5 molar equivalents of the non-esterified fatty acid palmitate (C16:0) reduced the Co^2+^-binding affinity at both sites A and B. The presence of bound myristate (C14:0) in the HSA crystal structures provided insight into the fatty acid-mediated structural changes that diminish the affinity of the protein toward Co^2+^. Together, these data provide further support for the idea that ischemia-modified albumin corresponds to albumin with excessive fatty-acid loading. Collectively, our findings provide a comprehensive understanding of the molecular underpinnings governing Co^2+^ binding to serum albumin.

## Abstract 2

Serum albumin–Co^2+^ interactions are of clinical importance.

## Graphical abstract

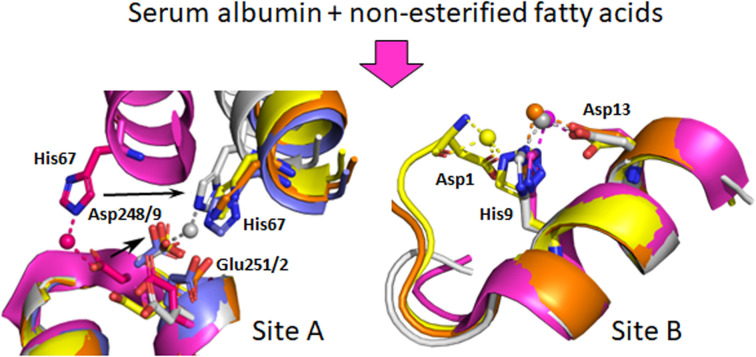

## 1. Introduction

Cobalt is an essential trace element in the body as a component of vitamin B_12_.^1^ However, cobalt in the ionic form Co^2+^ is a toxic substance. Co^2+^ is often present in the circulation at low plasma concentrations (around 0.2 μg L^−1^),^2,3^ where it interacts primarily with human serum albumin (HSA).^4^ Excessive cobalt levels (cobalt poisoning) can occur through over-consumption, inhalation or skin contact,^5^ and have been reported in individuals who have occupations where cobalt is frequently used, namely within settings that include cobalt processing plants, hard-metal industries and diamond polishing.^1,6^ Additionally, the use of metal-on-metal (MoM) joint implants (containing cobalt-chromium alloys) in orthopaedic joint replacements has been suggested to lead to high circulating cobalt levels in patients with such implants.^7^ Indeed, Co^2+^-toxicity, which can result in neurological, cardiovascular and endocrine symptoms,^8^ often occurs in such patients.^9^ It has also been suggested that elevated circulating cobalt levels can cause inflammatory and hypersensitive reactions^10^ and that Co^2+^ binding to HSA may form haptens that trigger autoimmune responses.^11^ In addition, Co^2+^–HSA interactions are important for the early diagnosis of myocardial ischemia. A reduced binding capability of HSA for Co^2+^ was seen in 96% patients with myocardial ischemia.^12^ An albumin-cobalt-binding (ACB) assay, the only available method for detecting the plasma biomarker ischemia-modified albumin (IMA), was developed to rapidly diagnose this condition. However, the usefulness and accuracy of this test are questionable due to many other conditions eliciting a positive test, which leads to high rates of false positive results. There is therefore a requirement for the Co^2+^-binding properties of HSA to be fully explored.

HSA is the most abundant protein in plasma (*ca.* 600–700 μM) and is the main carrier for Co^2+^ when present in the circulation.^13,14^ HSA is also capable of binding non-esterified fatty acids (NEFAs),^15^ hormones and drugs, as well as a variety of other metal ions, including Zn^2+^, Ca^2+^, Cd^2+^, Cu^2+^, Mg^2+^, Mn^2+^ and Ni^2+^.^16,17^ Based on spectroscopic studies and affinity measurements, three Co^2+^-binding sites have been proposed to exist in HSA and other mammalian albumins: (1) the amino terminal Cu- and Ni-binding (ATCUN) motif (also called the N-terminal binding site or NTS). ATCUN is composed of the first three amino acids (Asp1-Ala2-His3 in HSA); (2) site A (also known as the multi-metal binding site or MBS), which includes His67 (in domain I), His247 and Asp249 (in domain II);^18^ (3) Site B, proposed to include His9, Asp13 (in domain I) and, more tentatively, Asp255 (in domain II).^19^ In addition, Cys34, the fourth major metal ion binding site found in HSA, which binds Au^+^ and Pt^2+^ complexes,^20,21^ has been ruled out as Co^2+^ binding site.^22^ The NTS was the first site to be proposed as the primary Co^2+^-binding site, based on ^1^H-NMR studies that indicated perturbation of resonances for the three N-terminal residues on HSA upon addition of Co^2+^.^23^ Furthermore, a study conducted using synthetic peptides also implied that the N-terminus could be a Co^2+^-binding site on HSA.^24^ Bar-Or *et al.* proposed that HSA from myocardial ischemia patients may possess N-terminal modifications resulting in lower Co^2+^ binding.^12^ Based on this hypothesis, the ACB assay was developed,^25^ which was later approved by the FDA. However, strong evidence obtained from electronic absorption spectroscopy, CD spectroscopy, ^1^H-NMR, ^111/113^Cd-NMR and isothermal titration calorimetry (ITC) experiments indicated that sites A and B are the principal binding sites for Co^2+^ rather than the NTS.^22,26,27^ It has also been reported that the binding site preference for Co^2+^ is site B > site A > NTS, with *K*_D_ values of 11 μM, 90 μM and 110 μM, respectively.^26^

We have since proposed that elevated NEFAs, allosteric regulators of Zn^2+^ binding at site A, may also be responsible for reduced Co^2+^ binding in myocardial ischemia patients.^27^ Current evidence indicates that NEFA-binding to bovine serum albumin (BSA) leads to a structural change that diminishes Co^2+^ binding, with potentially both sites A and B affected.^28^ Among seven well-characterised NEFA-binding sites (FA1-7),^29–31^ FA2 is of particular interest as it is located between sub-domain IA and IIA, near to site A. Our previous NMR and ITC studies have revealed that the Zn^2+^-binding capacity of mammalian serum albumins is significantly perturbed by the presence of medium-to long-chain NEFAs, as their binding at FA2 results in an allosteric rearrangement of metal-coordinating residues in site A.^32–34^ However, similar work has not yet been conducted relating to Co^2+^ binding to HSA, and no structural data are available for any mammalian albumin–Co^2+^-complex.

In this study, we elucidate the X-ray crystal structures of HSA and equine serum albumin (ESA) in complex with Co^2+^ to determine location and structures of Co^2+^-binding sites on serum albumins. HSA and ESA are composed of 585 and 583 amino acids, respectively, and share 76.1% sequence identity.^19,35^ In general, the structures of ESA and HSA are highly similar; both are composed of three homologous domains that form a “heart” shape, have 17 conserved disulfide bonds and possess a single free thiol group at Cys34.^19,35,36^ We also unequivocally identify the major Co^2+^-binding sites on HSA using site-directed mutagenesis and examine the effects of NEFA binding (palmitate) on Co^2+^-binding to both wild-type HSA and mutated HSA forms.

## 2. Experimental

### 2.1. Materials for crystallisation

HSA and ESA isolated from natural sources were purchased from Sigma-Aldrich (St. Louis, MO, USA; A3782-1G) and Equitech-Bio (Kerrville, TX, USA; #ESA62), respectively as lyophilised powder and purified further as described below. The final protein purity was above 99% and 95% for HSA and ESA, respectively, as assessed by SDS-PAGE and gel filtration chromatography. Cobalt(ii) chloride (#C3169) and myristic acid (#M3128) were purchased from Sigma-Aldrich.

### 2.2. HSA crystallisation

HSA was dissolved in gel filtration buffer 1 (25 mM Tris, 50 mM NaCl, pH 7.4) and further purified by gel filtration chromatography using this buffer in the mobile phase on a Superdex 200 column attached to an ÄKTAprime (Cytiva, Chicago, IL, USA) at 4 °C. The HSA concentration was estimated spectrophotometrically by measuring the absorbance at 280 nm with a Nanodrop 2000 (Thermo Fisher Scientific, Waltham, MA, USA) and using the extinction coefficient *ε*_280-HSA_ = 34 440 M^−1^ cm^−1^ and molecular weight MW_HSA_ = 66 470 Da. The collected fractions of monomeric HSA were combined and concentrated to 88–100 mg mL^−1^ using an Amicon Ultra Centrifugal Filter (Sigma-Aldrich, #UFC903024) with a 30 kDa molecular weight cut-off. The concentrated protein was incubated at 37 °C while 2 μL aliquots of 500 mM myristic acid dissolved in ethanol were added and allowed to incubate for 30 minutes until the additional portion left the solution saturated. The excess myristic acid in form of a white solid was removed using Ultrafree-MC GV Centrifugal Filters (Merck Millipore Ltd.) with pore size of 0.22 μm. Protein crystallisation was performed in 96-well sitting-drop plates (Swissci 96-Well 3-Drop Plates, SWISSCI, Neuheim, Zug, Switzerland). A Mosquito crystallisation robot (TTP Labtech, Boston, MA) was used to set up 200:200 nL crystallisation solution:albumin drops. Crystals grew in 1–2 days at 18 °C. For 8EW4 and 8EY5, initial crystals were found in solution D2 of the BCS Screen from Molecular Dimensions. For the data collection crystals, aliquots of 200 nL of 88 mg mL^−1^ HSA were mixed with 200 nL of solution (22.5% PEG smear low (PEGSL; Molecular Dimensions, Holland OH, USA), 10% isopropanol, 100 mM Tris, pH 7.4), and 50 nL of seeds, which were created by crushing initial crystals in buffer (45% PEGSL and 100 mM Tris, pH 7.4). The reservoirs contained 35 μL of 1.0 M NaCl. 400 nL of buffer with cobalt (20 mM CoCl_2_, 45% PEGSL 100 mM Tris, pH 7.4) were gently added to the drops with crystals 9 hours prior to harvesting to reach a final concentration of ∼9 mM CoCl_2_. For the 8EW7 data collection crystal, 100 mg mL^−1^ HSA saturated with myristic acid was diluted with buffer 1 to 50 mg mL^−1^, aliquots of 200 nL of protein sample were mixed with 200 nL reservoir solution (18% PEG3350, 50 mM Tris, pH 7.4) and 50 nL of seeds suspended in buffer (40% PEG3350, 50 mM Tris, pH 7.4). A few days before harvesting, 2 μL of buffer with cobalt (20 mM CoCl_2_, 40% PEG3350, 50 mM Tris, pH 7.4) was gently added to drops with crystals to reach a final concentration of ∼16 mM CoCl_2_. All crystals were flash cooled in liquid nitrogen directly from the soaking drops.

### 2.3. ESA crystallisation

For purification, ESA was dissolved in gel filtration buffer 2 (10 mM Tris, 150 mM NaCl, pH 7.4) and further purified by gel filtration chromatography using this buffer in the mobile phase on a Superdex 200 column attached to an ÄKTAprime (Cytiva, Chicago, IL, USA) at 4 °C. The ESA concentration was estimated spectrophotometrically by measuring the absorbance at 280 nm with a Nanodrop 2000 using the extinction coefficient *ε*_280-ESA_ = 27 400 M^−1^ cm^−1^ and molecular weight MW_ESA_ = 65 700 Da. Collected fractions of monomeric ESA were combined and concentrated to 34 mg mL^−1^ using an Amicon Ultra Centrifugal Filter with a 30 kDa molecular weight cut-off. Protein crystallisation was performed in 15-well hanging drop plates (EasyXtal 15-Well Tools, Qiagen, Germantown, MD, USA). Aliquots of 1 μL of 34 mg mL^−1^ ESA were mixed with 1 μL of reservoir solution (0.2 M lithium sulfate, 2.0 M ammonium sulfate, 0.1 M Tris, pH 7.4). CoCl_2_ solution (3.3 μL of 50 mM) dissolved in the reservoir solution was added directly to each 2 μL crystallisation drop containing crystals to reach a final cobalt concentration of 31 mM and then incubated for 3–4 hours before harvesting. Harvested crystals were flash cooled in liquid nitrogen using Paratone N as a cryoprotectant.

### 2.4. Data collection and structure determination

Data collection was performed using single crystals at the Structural Biology Center's beamlines 19-BM and 19-ID and at the Life Sciences Collaborative Access Team beamline 21-ID-D at the Advanced Photon Source, Argonne National Laboratory (Argonne, IL, USA). The experiment was performed at 100 K. For HSA, X-rays with a wavelength of 1.604 Å (above the cobalt K-edge) were used to observe an anomalous signal from cobalt and the data were collected at 1.625 Å (below edge) to verify the loss of the anomalous signal. This can confirm with high certainty that cobalt was responsible for the observed anomalous signals. For ESA, a wavelength of 0.979 Å was used. HKL-3000 (ref. 37 and 38) was used to process, integrate, and scale the data. Corrections for radiation decay and anisotropic diffraction were applied.^39^ Before data collection, the presence of cobalt in the ESA crystal was confirmed by a fluorescence spectrum with the excitation energy at 12 664 eV (0.979 Å) using HKL-3000.^40^ In structures of HSA (PDB ID: 6WUW)^41^ and ESA (PDB ID: 3V08)^35^ were used as templates for molecular replacement. Structure determination and refinement were performed using HKL-3000 integrated with MOLREP,^42^ REFMAC,^43^ Coot,^44^ and other programs from the CCP4 package.^45^ The refinement process followed recent state-of-the-art guidelines.^46,47^ TLS groups, determined by the TLS Motion Determination Server, were applied during refinement.^48^ The ACHESYM server^49^ was used for the standardised placement of the model in the unit cell. PyMOL (The PyMOL Molecular Graphics System, Version 1.5.0.3 Schrödinger, LLC) and ChemSketch were used for figure generation. The Dali server^50^ was used for structure comparison and calculation of Cα RMSDs. The CheckMyMetal server was used to validate the quality of the modelled metal-binding sites.^51^ Molstack was used for the creation of an interactive model and map representation.^52,53^ A comparison of the structures presented here, including above- and below-the-edge anomalous maps, is accessible at https://molstack.bioreproducibility.org/project/view/6DHigoArQCu5TdDAfzBj/. The statistics for diffraction data collection, structure refinement, and structure quality are summarised in Table 1. Diffraction images are available at the Integrated Resource for Reproducibility in Macromolecular Crystallography at http://proteindiffraction.org.^54^ The atomic coordinates and structure factors were deposited in the RCSB PDB with accession codes 8EW4, 8EW7, 8EY5 for HSA and 7MBL for ESA.

**Table 1:** Data collection, structure refinement, and structure quality statistics. Values in parentheses are for the highest resolution shell. Ramachandran plot statistics are calculated by MolProbity

PDB ID	7MBL	8EW4	8EW7	8EY5
Diffraction images DOI	10.18430/m3.irrmc.5869	10.18430/M38EW4	10.18430/M38EW7	10.18430/M38EY5
Resolution (Å)	50.00–2.70 (2.75–2.70)	40.00–2.40 (2.44–2.40)	40.00–3.30 (3.36–3.30)	40.00–3.10 (3.15–3.10)
Wavelength (Å)	0.979	1.604	1.603	1.604
Space group	*P*6_1_	*C*2	*C*2	*C*2
Unit-cell dimensions: *a*, *b*, *c* (Å)	93.3, 93.3, 141.5	172.8, 38.5, 95.9	166.1, 38.3, 96.7	186.0, 38.6, 93.4
Angles: α, β, γ (°)	90.0, 90.0, 120.0	90.0, 103.6, 90.0	90.0, 105.0, 90.0	90.0, 103.8, 90.0
Protein chains in the ASU	1	1	1	1
Completeness (%)	92.9 (100.0)	92.2 (93.5)	93.2 (100.0)	93.7 (99.5)
Number of unique reflections	19 239	24 101	9385	12 191
Redundancy	5.0 (5.1)	3.3 (3.2)	6.6 (6.6)	3.4 (3.4)
<*I*>/<*σ*(*I*)>	17.3 (1.4)	18.5 (4.0)	14.9 (3.4)	20.8 (3.3)
CC 1/2	(0.74)	(0.95)	(0.93)	(0.85)
Rmerge	0.095 (1.129)	0.061 (0.283)	0.127 (0.565)	0.057 (0.446)
Rpim	0.047 (0.560)	0.039 (0.180)	0.054 (0.238)	0.036 (0.283)
Rwork/Rfree	0.220/0.287	0.209/0.266	0.194/0.272	0.205/0.260
Bond lengths RMSD (Å)	0.007	0.003	0.003	0.003
Bond angles RMSD (°)	1.3	0.9	1.0	1.0
Mean ADP (Å^2^)	58	41	46	64
Mean ADP for ligands/ions (Å^2^)	91	42	52	60
Number of protein atoms	4582	4643	4621	4622
Mean ADP for protein (Å^2^)	58	41	46	65
Number of water molecules	67	161	44	43
Mean ADP for water molecules (Å^2^)	61	34	20	37
Clashscore	5.0	2.5	5.3	4.3
MolProbity score	1.7	1.0	1.6	1.3
Sidechain outliers (%)	2.2	0.6	1.2	1.2
Ramachandran outliers (%)	0.0	0.0	0.0	0.0
Ramachandran favored (%)	97.4	98.3	97.6	99.14

### 2.5. HSA mutagenesis, expression and purification

A synthetic gene containing the coding sequence of HSA (corresponding to residues 25-609 of the albumin preprotein sequence; NP_000468.1) was purchased from Eurofins Genomics (Ebersberg, Germany). The sequence was amplified and cloned into pEX-A258 vector (Thermo Fisher Scientific). The constructed plasmid (pEX-A258-HSA) was digested using *Xho*/*NotI* restriction enzymes and the targeted HSA gene was ligated into pPICZαB vector in frame and immediately downstream from the region encoding the α-factor secretion signal sequence. The recombinant plasmid was transformed into TOP10F′ *E. coli* cells according to the manufacturer's protocol (Invitrogen, USA). Oligonucleotide directed mutagenesis was used to prepare constructs encoding mutated albumins (H3A, H9A, H67A, H247A and H9A/H67A) using the GENEART™ Site-Directed Mutagenesis System (Thermo Fisher Scientific, UK; #A13282). The resultant plasmids were linearised using *PmeI* enzyme and transformed into DH5α™-T1^R^*E. coli* competent cells under selection by 25 μg mL^−1^ zeocin (InvivoGen, Toulouse, France; #ant-zn-5b). All plasmids were isolated and confirmed by restriction analysis and DNA sequencing. After confirmation, plasmid DNA from resultant clones were extracted and transformed into X-33 *Pichia pastoris* competent cells by electroporation. Successful integration into the *Pichia pastoris* genome was assessed by PCR, using primers flanking the insertion site. In the case of protein expression, a colony was grown overnight in 15 mL of sterile buffered glycerol-complex (BMGY) medium (1% yeast extract, 2% peptone, 1.34% yeast nitrogen base, 0.4 mg L^−1^ biotin, 1% glycerol, 100 mM potassium phosphate, pH 6.0) containing 100 μg mL^−1^ zeocin at 28 °C with shaking at 200 rpm. This seed culture was inoculated in 350 mL of BMGY medium and grown overnight until the culture reached log phase growth (OD_600_ = 2–6). Cells were harvested and resuspended in 1 L of sterile BMGY medium. The culture was grown at 28 °C for 5 days with shaking at 200 rpm and protein was expressed with addition of 0.5% methanol every 24 hours. Finally, the supernatant containing the protein of interest was obtained by centrifuging at 8000 × *g* for 30 minutes at 4 °C. A concentrated sample of supernatant was loaded onto a HiTrap Blue HP column (Cytiva, Buckinghamshire, UK; #17041301) pre-equilibrated with binding buffer (20 mM potassium phosphate, pH 7.0). After washing, protein of interest was eluted with buffer containing 1.5 M KCl, 20 mM potassium phosphate, pH 7.0. Final purity of the protein was verified by SDS-PAGE (estimated to be >95%). Protein sequence and intact mass were also confirmed by mass spectrometry. Proteins were dialysed in desired buffer prior to downstream experiments.

### 2.6. ^1^H-NMR spectroscopy

Structural integrity of mutant forms of HSA was confirmed by ^1^H-NMR spectroscopy, using 50 μM protein samples in 10 mM sodium phosphate, 140 mM NaCl, 2% (v/v) D_2_O, at pH 7.4. One-dimensional ^1^H-NMR spectra with water suppression by excitation sculpting (Bruker pulse sequence zgesgp) were recorded with 256 scans at a spectral resolution of 1.7 Hz on a Bruker Ascend 700 MHz spectrometer equipped with a Prodigy TCI probe at 22 °C. The spectra were processed and analysed using Bruker Topspin 4 (RRID:SCR_014227).

### 2.7. Isothermal titration calorimetry (ITC)

The binding of Co^2+^ to commercial HSA purified from human plasma (HSA) (Sigma-Aldrich, Poole, UK; #A1887), ESA (Equitech-Bio; #ESA62) or recombinant HSA forms was analysed by ITC using a MicroCal VP-ITC instrument (Malvern, UK). Proteins and cobalt(ii) chloride (Sigma-Aldrich, UK; #C8661) were prepared with the same buffer (50 mM Tris, 50 mM NaCl, pH 7.4). Sodium palmitate (Sigma-Aldrich, UK; #P9767) was prepared in methanol. All samples were degassed under vacuum prior to use. In the cell, proteins with a concentration of 50 μM (in the presence of 0 or 5 molar equivalents (mol. eq.) of palmitate) were titrated with 2 mM cobalt(ii) chloride. Each experiment was performed at 25 °C with stirring speed at 307 rpm. The parameters were set to provide a first injection of 2 μL over 4 s followed by up to 35 total injections of 8 μL over 16 s each with an interval of 210 s to allow complete equilibration between injections. Blank titrations to account for heats of dilution were performed by titrating cobalt(ii) chloride solution into buffer. The resultant data representing the averaged heat of dilution were subtracted from the data obtained from the main experiments. Raw data were obtained using MicroCal Origin 7.0 software. AFFINImeter software (Santiago de Compostela, Spain) was additionally employed to fit the data (Table S1†). Fitting quality was evaluated by the goodness of fit (0.7 < *χ*^2^ < 3). Data presented are representative of at least 3 experiments.

### 2.8. Circular dichroism (CD)

Circular dichroism experiments were performed using a MOS-500 spectropolarimeter from BioLogic (Seyssinet-Pariset, France). Samples contained 0.5 mM protein in 100 mM HEPES, pH 7.4. In each case, small volumes of 1.5 M cobalt(ii) chloride (prepared in Milli-Q water) were titrated into the mixtures. Spectra were recorded at 25 °C in the range of 240 to 800 nm in 1 cm cuvettes. Due to the negligible total volume changes, data were not corrected for dilution. The spectrum of buffer (blank) was subtracted from each spectrum. A spectrum obtained of protein without Co^2+^ was also subtracted and final spectra normalised to baseline values. The final data were plotted and fitted using Graphpad Prism.

## 3. Results and discussion

### 3.1. Structural characterisation of Co^2+^ complexes with HSA and ESA

**Fig. 1:**
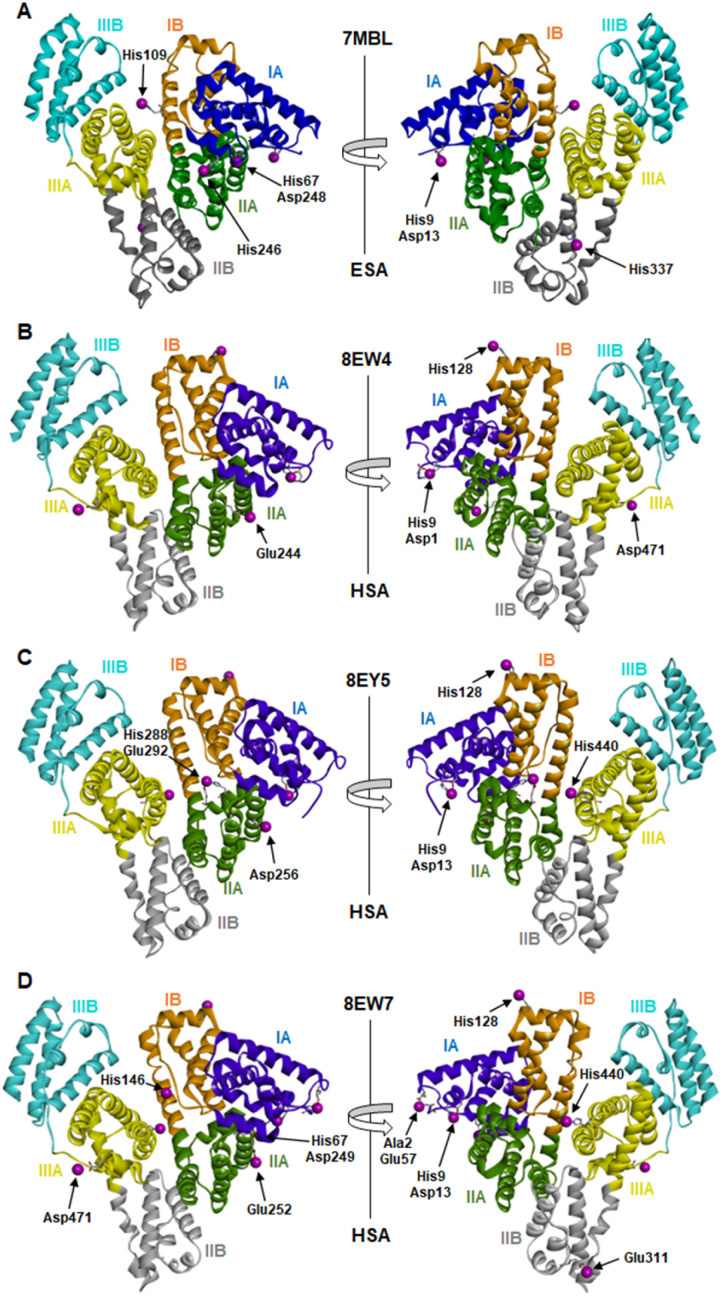
Locations of cobalt binding sites in the ESA and HSA structures. A, B, C and D correspond to PDB IDs 7MBL, 8EW4, 8EY5 and 8EW7, respectively. Cobalt ions are shown as purple spheres. Residues belonging to the first coordination spheres are shown as sticks and labelled, each on only one panel. Domains are labelled with Roman numerals (I, II, III) and subdomains with letters (*e.g.*, IB), with each subdomain shown in a different colour (as indicated).

The locations and compositions of Co^2+^-binding sites on serum albumins have not previously been structurally characterised, with the site preferences described above inferred from spectroscopic and calorimetric measurements. Here we obtained four Co^2+^–albumin structures representing structures of the protein from both human (8EW4, 8EW7 and 8EY5) and equine species (7MBL). A summary of these four Co^2+^–albumin crystal structures with Co^2+^-binding sites labelled is presented in Fig. 1. To maximise the reliability of our findings, the structures from four crystals of HSA with bound myristate soaked in 9–16 mM CoCl_2_ solutions at resolutions of 2.4–3.3 Å were determined (data accessible *via* molstack; see Section 2.4). Note that one of these structures is not presented here as it resembled 8EY5 but had poorer R and Rfree values and was of lower resolution. It is available *via* the molstack link above. Crystals of HSA without myristate bound were also obtained but were too fragile for soaking. Attempts to soak these crystals with cobalt(ii)-containing solutions led to cracking. This was much less common with crystals of HSA where myristate was bound. Due to radiation damage, only the crystal for 8EW4 was in a condition that allowed performing a second data collection below edge, where the anomalous signal in the site involving both His9 and Asp1 dropped from 16.9 to the noise level of 2.0 RMSD, confirming the presence of Co^2+^ at this location. Thus, cobalt atoms were modelled in the potential metal binding sites (data shown in molstack). In the case of ESA, a total of seven structures from crystals soaked with CoCl_2_ solutions at resolutions of 2.3–2.8 Å were determined and examined (data not shown for six crystal structures). The presence of cobalt in the ESA crystals was confirmed by fluorescence spectra with the excitation energy at 12 664 eV (0.979 Å) using HKL-3000, which showed characteristic peaks for cobalt. Structure 7MBL was chosen as the representative dataset with the highest resolution that showed anomalous signals for all five Co^2+^ sites observed across the ESA structures examined.

It is noteworthy that a soaking approach, rather than co-crystallisation, was adopted due to the high potential for Co(ii) to oxidise to Co(iii) over the required timescale for crystals to form.^22^ Based on previous observations with Zn^2+^,^19^ putative metal-binding sites on albumin are essentially pre-formed, indicating that major conformational changes are not expected to be required to facilitate metal binding. The crystals of HSA in complex with Co^2+^ belong to the *C*2 space group, while the ESA crystal structure presented belongs to the *P*6_1_ space group. All structures contain one protein chain in the asymmetric unit. The protein models are complete except for the first two N-terminal residues in 7MBL, the last C-terminal residue in 8EW4, 8EW7, and 8EY5 and the first N-terminal residue in 8EW7, for which the electron density was not observed. This is a common occurrence in serum albumin structures due to flexibility in the N-terminal and C-terminal regions.^19,55,56^ The overall fold and conformation of the HSA and ESA molecules resemble structures that have been reported before.^19,36,57,58^ Indeed, the structure of ESA (7MBL) adopts a highly similar fold to HSA structures that do not possess bound NEFA molecules (*e.g.* 1BM0, Fig. S1A†),^56^ while the three HSA structures, all of which contain bound myristate, adopt conformations similar to published structures with palmitate or myristate bound (1E7H and 1BJ5, respectively; Fig. S1B†).^31,58^ Crucially, this means that the soaking process did not significantly affect any NEFA-dependent differences in overall conformation.

Myristate binding at up to ten sites in the HSA structures was observed and is summarised in Table S2.† The structure of 8EW4 in complex with this NEFA (representing the fully saturated form), showing the locations of binding sites, is presented in Fig. S2.† The crystal conditions used for 8EW7 involved co-crystallisation with half the concentration of myristate of that used for 8EW4 and 8EY5, and a five times larger volume of soaking solution was used which further diluted the concentration of myristate in the crystallisation drop. We estimate that the concentration was around 6-times lower than in the other two HSA structures, although due to equilibration of crystal drop-reservoir this value is not precisely known. This explains the lower number of NEFA molecules bound in this structure. The latter conclusion is supported by analysis of the FA1 site (Fig. S3†). In 8EW7 the side chains of Tyr138 and Tyr161 are in the space where myristate is known to bind (indeed, in 8EW4 these two residues are in a different position to make space for bound myristate),^31^ confirming that at the time of harvesting, the crystal for 8EW7 possessed a lower number of bound myristate molecules than for example 8EW4, and that the unoccupied sites are not a result of low crystallographic resolution.

In the NEFA-bound HSA structures, there are two regions that exhibit significantly lower RMSD when locally superimposed with the unliganded ESA structure, as compared to globally aligned structures. These regions are residues 1–185 (roughly corresponding to domain I) and 210–585 (part of domain II and all of domain III). The alpha-helix located between residues 173–206 acts like a “hinge” to facilitate a switch between the two conformations (Fig. S4A†). We locally superimposed fatty acid binding sites of both 7MBL and 8EW4 structures, and in all cases the main chain does not exhibit significant steric clashes with myristate except in FA2 (as can be seen in Fig. S4B–E†). Thus, as previously observed,^31^ the FA2 site only becomes available for binding medium- and long-chain NEFAs after a repositioning of the domains. In previously published structures, NEFAs in this binding site are bound in two half-pockets, formed by Arg10, Leu14, Phe19, Leu22, Val23, Ala26, and Leu66 from domain I and Leu250, Leu251, Ala254, Ala258, Leu283, and Leu284 from domain II, with the carboxylate headgroup interacting with Tyr150, Arg257 and Ser287.

Surprisingly however, even though our NEFA-loaded HSA structures show conformations close to those of previously published structures in complex with NEFAs, two of them (8EW7 and 8EY5) did not show sufficient electron density for myristate to be modelled into FA2. In 8EW4, a myristate molecule is present in/near FA2, albeit with a different conformation and placement in comparison to that observed in 1BJ5:^58^ in 8EW4 the myristate is displaced by 6.4 Å within one half-pocket relative to its position in 1BJ5, with the carboxylate headgroup buried less deeply in this half-pocket and making contact with Arg10 instead of Tyr150, Arg257 and Ser287 (Fig. S5†). Essentially, the front half of myristate in 8EW4 is in a similar location as the tail end in 1BJ5, whilst the tail end in 8EW4 is located between the two domains. We also cross-superposed both placements of myristate in 1BJ5 and 8EW4, and in both cases there were no significant steric clashes, meaning that the “conventional” FA2 binding pockets are still present in 8EW4. The new orientation is evocative of this NEFA molecule being in the course of leaving its binding site. We suggest that the soaking process may promote the loss of myristate from this site, explaining not only the new position in 8EW4 but also the absence of myristate in 8EW7 and 8EY5 – whilst nonetheless leaving the protein in a locked conformation.^30,59^ It is unclear why site FA2 (which is considered a high-affinity site^30^) in particular was affected in this way, but most importantly for the following discussion, the Co^2+^ ions encountered albumin in a NEFA-induced conformation.

### 3.2. Detection and identification of Co^2+^-binding sites

The anomalous map peaks indicated 16 locations for bound Co^2+^ across the four presented structures where coordinating residues were sufficiently ordered to be modelled (Table 2). These represent 5 sites on ESA and 13 sites on HSA. The only cobalt-bound side-chain common to all four structures is His9, proposed to be a ligand in site B. Residues from adjacent protein chains, which could potentially influence binding in the crystal are also indicated. For the HSA structures the strength of the anomalous peaks ranged from 3.5 to 18.4 RMSD. Even though we collected data above and below edge only for the 8EW4 structure, we modelled Co^2+^ ions also for the other structures in the positions that had a significant anomalous signal, because the only ions that were present in our systems were Na^+^ and Co^2+^, and only Co^2+^ can produce an anomalous signal at this wavelength. For ESA, anomalous peaks were observed for each of the five sites in at least three (out of seven) structures. The strength of the anomalous peaks ranged from 3.0 to 6.3 RMSD (3.0–4.7 RMSD in the structure presented). Importantly, the heights of the peaks should not be directly used to indicate which site is stronger because the strength of the anomalous peaks is a function of both metal occupancy and the static disorder (difference between individual molecules within the crystal) and because crystals were saturated with cobalt.

**Table 2:** Summary of experimental conditions and Co^2+^-binding sites in X-ray crystal structures presented. The presence of specific sites is indicated by “x”. Residues from adjacent protein chains within the crystal lattice that reside close enough to site to potentially influence binding are provided in italics. “a” indicates where such a residue is < 6 Å but >3.2 Å from the cobalt atom and “b” where it is ≤3.2 Å from the cobalt atom. “†” denotes interaction with a side chain. “‡” denotes interaction with the main chain (for example, carbonyl oxygen)

PDB ID		7MBL	8EW4	8EY5	8EW7
Albumin		ESA	HSA	HSA	HSA
Co^2+^ concentration		31 mM	9 mM	9 mM	16 mM
Duration of soaking		3–4 h	9 h	9 h	Several days
Site no.	Co-ordinating residues				
I	His9, Asp13	×		×	×
II	His9, Asp1		×		
III	His67, Asp248	×			
III'	His67, Asp249				×^a^
					†*Glu227*
IV	Ala2, His3, Glu57				×^a^
					†*Glu358*, †*Lys323*
V	His109	×			
VI	His146				×
VII	His288, Glu292			×	
VIII	His440			×	×
IX	His246	×^a^			
		†*Glu396*			
		†*Lys544*			
		†*Val397*			
X	His337	×^a^			
		†*Glu56*			
XI	His128		×^a^	×^b^	×^a^
			†*Glu119*, ‡*Arg117*	†*Glu119,* ‡*Arg117*, ‡*Pro118*	†*Glu119,* †*Glu520,* ‡*Arg117*
XII	Asp471		×^b^		×^a^
			†*Glu396*, ‡*Cys438*, †*Arg445*		†*Glu396*
					†*Arg445*
XIII	Glu244		×^b^		
			†*Ser270*, †*Glu230*, †*Asp269*, †*Asn267*		
XIV	Asp256			×^a^	
				‡*Glu266*, †*Asn267*	
XV	Glu252				×^a^
					†*Glu230*, †*Ser270*, †*Glu227*
XVI	Glu311				×^a^
					†*Gln397*

**Fig. 2:**
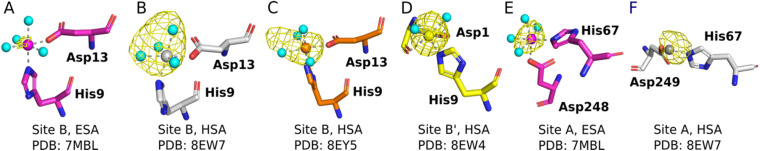
Structures and anomalous signals (RMSD = 3.0) in site B/B′ (residues Asp13/Asp1 and His9) and site A (residues His67 and Asp249/Asp248). Cobalt ions (central spheres) and carbons are coloured by structure: 7MBL, magenta; 8EW4, yellow; 8EW7, white; 8EY5, orange. (A) Site B of 7MBL. (B) Site B of 8EW7. (C) Site B of 8EY5. (D) Site B′ of 8EW7. (E) Site A of 7MBL. (F) Site A of 8EW7. Water molecules for sites where full octahedral geometries could be modelled are shown as cyan spheres.

The sites identified included Co^2+^ bound to site B (His9 and Asp13; Fig. 2A–C) or a variation of this site which will be referred to as site B′ (His9 and Asp1; Fig. 2D) and to site A (His67 and Asp248/9; Fig. 2E and F) in ESA and one HSA structure (8EW7). The shape of the observed omit map peaks suggested a broadly octahedral coordination for site B in 7MBL, 8EY5 and 8EW7, site B′ in 8EW4 and for site A in 7MBL, as expected for Co^2+^ in hard/intermediate sites.^60^ Consequently, these cobalt sites were modelled in a complete octahedral geometry with water molecules completing the coordination sphere. However, in site A of 8EW7, although the anomalous signal ensures Co^2+^ placement, there is insufficient density to confidently model a fully occupied octahedral geometry, and this was also the case for some other cobalt sites (Table 2). We note that owing to the relatively low resolution typical for albumin crystal structures, it was not possible to separate the electron density peaks for the metal and the coordinating water molecules; the geometric arrangement and cobalt-water distances obtained were mostly defined by the restraints applied during refinement. Theoretically, Co^2+^ also can adopt lower coordination numbers including tetrahedral geometries like those observed for Zn^2+^ bound to HSA sites, especially if most of the coordinating water molecules are replaced by larger substituents such as Cl^−^ ions.^61^ Approximately 75 mM of NaCl was present in the crystallisation drops prior to the addition of the CoCl_2_ solution, suggesting that some fraction of the first solvation sphere for cobalt could be replaced by Cl^−^ ions. Therefore, involvement of Cl^−^ ions and geometries involving lower coordination numbers cannot be excluded.

**Fig. 3:**
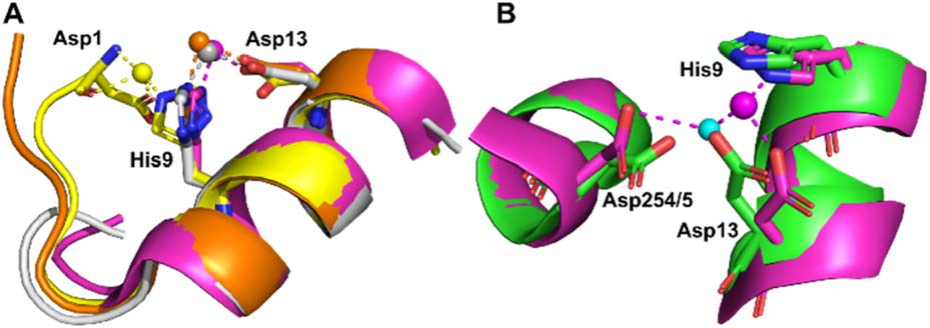
Structures of Co^2+^ binding sites B and B′. (A) An overlay of the four presented structures showing amino acid sidechains involved in Co^2+^-coordination at sites B and B′. The positions of Asp1, His9 and Asp13 in stick representation are shown (where present). In three of the structures Co^2+^ is co-ordinated *via* Asp13 and His9 (site B). In 8EW4, Asp1 takes the place of Asp13 providing 3 coordinating bonds for Co^2+^-binding (site B′). This is possibly due to the nearby location of a neighbouring protein chain in the crystal (which was absent in the other crystals) that alters the conformation of the N-terminus in this structure compared to the others (shown in Fig. S6A†). Co^2+^ ions (spheres) and carbons are coloured by structure: 7MBL (magenta); 8EW4 (yellow); 8EW7 (white); 8EY5 (orange). (B) structural comparison of site B side-chains of 7MBL (magenta) and 1BM0 (in green). Asp254/5 coordinates Co^2+^ in outer sphere mode *via* water shown in cyan.

Co^2+^ was found to bind to site B (His9 and Asp13) in three of the structures across the two species. However, in 8EW4 a variation in this site (referred to as site B′) was observed whereby Co^2+^ was coordinated to His9 and Asp1 (rather than Asp13) (Fig. 3A), which could be due to the influence of a neighbouring protein chain in the crystal (Fig. S6A†). Of note, several of the coordinating water molecules interact with outer sphere residues in each of the sites. With respect to Co^2+^ binding to site B of ESA (7MBL), Asp254 (corresponding to Asp255 in HSA) coordinates in an outer-sphere mode to Co^2+^, *i.e. via* a water molecule (with a distance between Co^2+^ and the carboxylate oxygen of approximately 4.4 Å) (Fig. 3B).^19^ Additionally, the overlay of site B shown in Fig. 3B suggests that (i) the residues forming site B in native HSA (1BM0)^56^ and in the presented ESA structure (7MBL) are in identical positions in the two albumins from different species, and (ii) site B is indeed pre-formed and requires only slight adjustment of sidechain conformations to accommodate Co^2+^.

However, Asp255 in the HSA structures presented is too far away to engage in Co^2+^ binding even in an outer-sphere mode (>6 Å away), likely a consequence of the conformational differences between NEFA-free and NEFA-loaded albumin. In the presence of NEFA, His9 and Asp13 are displaced by 5.1–6.8 Å and 4.3–5.2 Å, respectively, away from their corresponding positions in 7MBL (Fig. S7†). The relative distances between Asp254/5 and of His9/Asp13 were compared and are shown in Table S5† (the distance between Asp1 and Asp254/5 upon fatty acid binding cannot be compared due to the lack of Asp1 in 7MBL).

Site A has been proposed to be the secondary Co^2+^-binding site by Sokołowska *et al.*^26^ Co^2+^ binding at this site was observed in the structures of 7MBL (ESA) and 8EW7 (HSA), with Co^2+^ bound to His67 and Asp248/9. In NEFA-free ESA, additionally Glu251 coordinates in outer-sphere mode, forming a hydrogen bond with one of the Co^2+^-bound water molecules. In 8EW7, Glu251 does not participate in Co^2+^-binding to site A; instead, a close residue from a symmetry mate was found to potentially assist (site III′ in Table 2 and Fig. S6B†).

Comparison between Zn^2+^- and Co^2+^-bound albumins highlights the dynamic nature of these metal-binding sites, perhaps not entirely appreciated previously. Notably, for both sites A and B, the binding modes found for Co^2+^ differ significantly from those observed for Zn^2+^. A fully formed Zn^2+^-binding site A involves also His246/7 in addition to His67 and Asp248/9, although alternative binding modes with His246 swung away and binding a second Zn^2+^ ion have also been observed in ESA when soaked with high Zn^2+^ concentrations.^19^ This was also the case for ESA soaked with Co^2+^ (IX in Table 2). Glu396 (ESA, 7MBL) from an adjacent protein chain within the crystal lattice was also found to potentially support Co^2+^ binding at site IX. Similarly in site B, Asp254 coordinated directly to Zn^2+^ in an ESA structure. These different binding modes are likely dictated by the metal ions' preferred coordination geometries (tetrahedral for Zn^2+^ and octahedral for the larger Co^2+^ ion) and binding preferences (outer-sphere binding is more common for Co^2+^), but it is remarkable that albumin can apparently adapt its two major metal-binding sites to accommodate both tetrahedral and octahedral geometries.

**Fig. 4:**
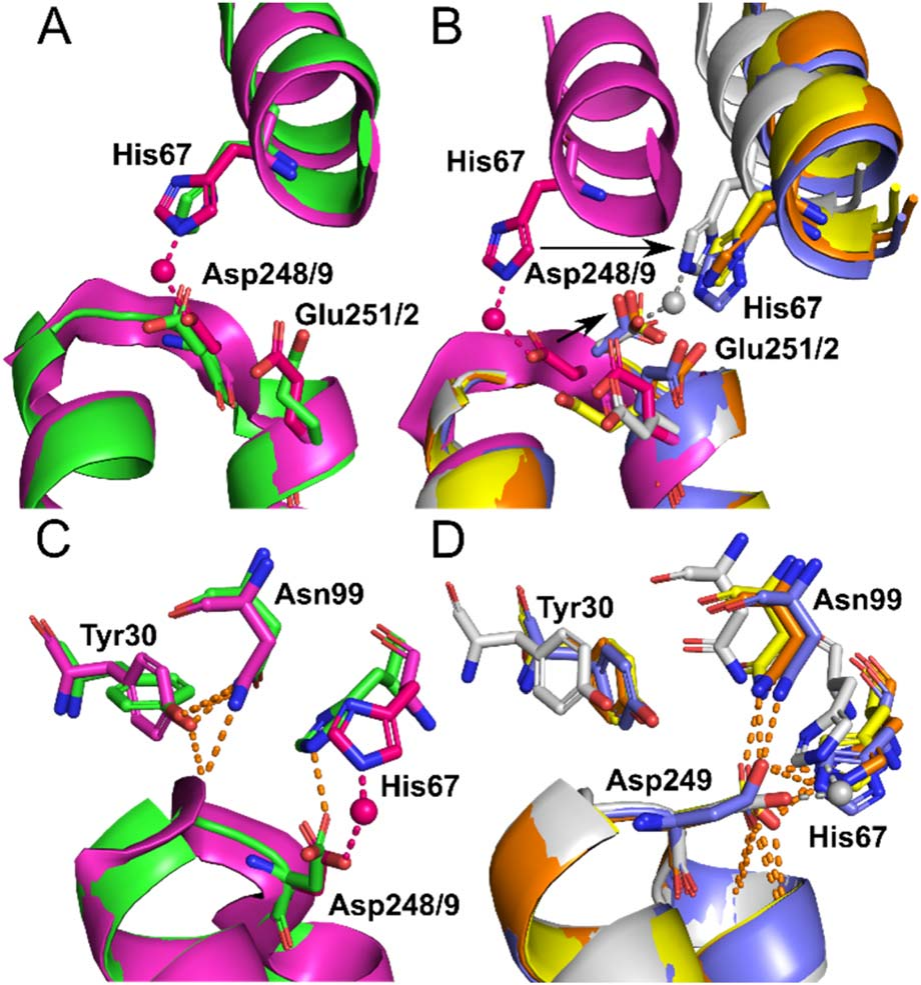
Structure of Co^2+^ site A. (A) Superimposition of fatty acid-free HSA structure (1BM0) in green with the presented fatty acid-free ESA–Co^2+^ structure (7MBL) in magenta. A high degree of similarity in the positions of the amino acids at this site is observed between serum albumin from the two mammalian species. The positions of His67, Asp248(ESA)/Asp249(HSA) and Glu251(ESA)/Glu252(HSA) in stick representation are shown. (B) Superimposition of the fatty acid-free ESA–Co^2+^ structure (7MBL) with the three myristate-bound HSA–Co^2+^ structures (8EW4 in yellow, 8EW7 in white and 8EY5 in orange) and myristate-bound HSA (1BJ5) in blue. A large structural alteration in this site occurs upon NEFA binding triggering a movement of the corresponding His and Asp residues, shown by black arrows. Due to lower concentration of fatty acid in solution, 8EW7 possesses a slightly different conformation of the main chain in comparison to fully saturated structures. (C) Superimposition of fatty acid-free HSA structure 1BM0 with the presented fatty acid-free ESA–Co^2+^ structure 7MBL. In this mode, His67 and Asp248/9 bind Co^2+^, while Asn99 interacts with the main chain and Tyr30, without interacting with Asn248/9. Mentioned sidechains are presented in stick representation. (D) Superimposition of the three myristate-bound HSA–Co^2+^ structures (8EW4, 8EW7, 8EY5) with the myristate-bound HSA structure (1BJ5). In this mode, Asp249 interacts with His67. Additionally, Asp249 interacts with Asn99 and the main chain in all cases except for 8EW7, due to a slight difference in conformation that allows the carboxylate group to rotate by 90° and interact with bound Co^2+^.

Identical conformations of site A sidechains were observed across unliganded HSA (1BM0) and Co^2+^–ESA (7MBL) as shown in Fig. 4A. As mentioned above, NEFA-binding facilitates a significant domain I-domain II movement, leading to a rearrangement of site A; His67 moves approximately 7.8 Å away relative to its initial position with the sidechain of Asp249 moving in the same direction (Fig. 4B). In the presented structures, only 8EW4 was fully saturated with myristate, with ten binding sites identified (Table S2 and Fig. S2†). Myristate was bound at nine and just three locations in 8EY5 and 8EW7, respectively. As discussed above, myristate could only be modelled in the FA2 site in 8EW4 and not in any of the other presented structures. Interestingly, the backbone of 8EW7 adopted a slightly different conformation than in the other two HSA structures where more myristate molecules were bound. This potentially allows the co-ordination of Co^2+^ at site A, albeit in a different manner to that seen in the NEFA-unbound state. Thus, what we see in 8EW7 appears to be a state between NEFA-unbound and NEFA-saturated states. We acknowledge that this “state” may be artefactual due to the presence of a myristate molecule in FA2 during crystallisation, which is subsequently lost through Co(ii) soaking. Under such conditions, the protein may be attempting to revert to the NEFA-unbound state but is prevented due to crystal contacts.

The different conformations observed in the HSA structures, where the relative positions of His67 and Asp249 vary, are likely to influence the affinity of Co^2+^-binding at this site. However, in each case it appears that these residues would be close enough to enable Co^2+^ coordination. The position of the Glu252 sidechain is altered in the NEFA-bound HSA structures (Fig. 4B). In 8EW4 and 8EW7 the sidechain points away from site A. Indeed, in 8EW7 it forms an additional single amino acid Co^2+^ site (site XV). In 8EY5, although Co^2+^ does not coordinate at site A, Glu252 lies in a conformation that could in principle provide an outer-sphere ligand. A clue as to why site A is not occupied by Co^2+^ in NEFA-bound 8EY5 and 8EW4 comes from examination of the H-bonding network at this region across available structures (Fig. 4C and D). In both structures, Asn99 forms an H-bond with Asp249 potentially stabilising the unbound state. In NEFA-free HSA and ESA structures Asn99 adopts a different position where it forms an H-bond with Tyr30, which may free up Asp249 to provide an O-ligand for Co^2+^ binding. The structure in 8EW7 is an exception – in this structure Asn99 adopts a position where it no longer creates an H-bond with Asp249, allowing this residue to rotate its carboxylate group to fit into the inner coordination sphere of Co^2+^.

Additionally, thirteen other Co^2+^-binding sites (sites IV to XVI) were found in the structures (Table 2). In these cases, the sites generally consisted either of a single amino acid sidechain and/or had the potential influence of a symmetry mate sidechain in aiding binding. Of eight of these sites involving histidine residues, six are formed by a single histidine (V, VI, VIII, IX, X and XI), one is formed by a single histidine and a single glutamate (VII), and one involves the backbone amide nitrogens of His3 and Ala2 and a glutamate (IV). It is worth noting that a 3.1–4.2 RMSD peak was also observed in the vicinity of His3 in most of the ESA structures; however, due to very high disorder (as illustrated in molstack), it was not possible to model cobalt with a complete coordination sphere, even though this residue has previously been implicated in Co^2+^ binding.^22,23,26^ Of the sites without histidine engagement, two are formed by a single aspartate (XII and XIV), three are formed by a single glutamate (XIII, XV and XVI), and one is formed by one alanine and one glutamate (XIV). It is worth noting that His109 in ESA (V) is not conserved in HSA, where the corresponding residue is an asparagine.^22,23,26^

### 3.3. Isothermal titration calorimetry of Co^2+^-binding to ESA and HSA

**Fig. 5:**
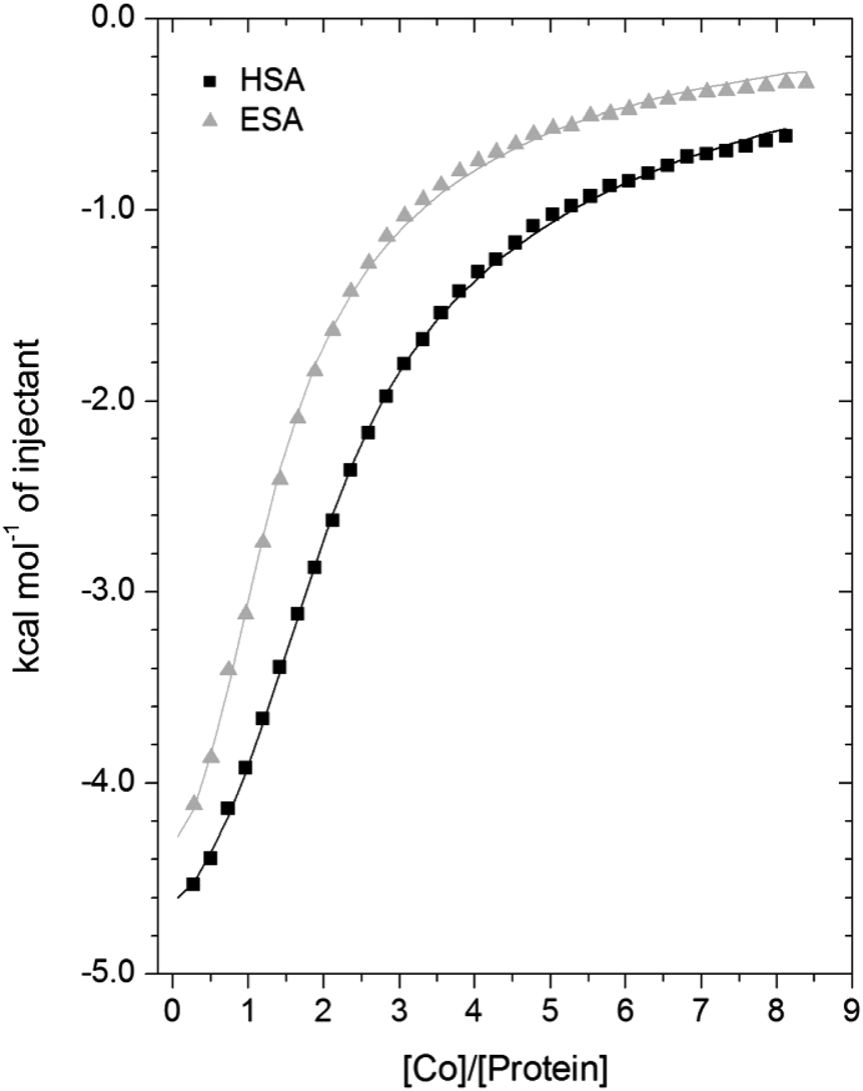
Measurement of Co^2+^ binding to plasma-purified HSA and ESA using ITC. In all cases, 50 μM of respective protein was titrated with 2 mM cobalt(ii) chloride solution for 35 injections. The data were fitted with a 3-sequential-sites model using MicroCal Origin 7.0 software (Fit 1–2).

A comparison of the Co^2+^-binding characteristics of ESA and HSA in solution was carried out using ITC (Fig. 5; raw data corresponding to the titrations shown are provided in Fig. S8 and S9†). An important aspect that determines the success of this approach is the concentrations of the protein and ligand in the experimental set-up. The appropriate protein concentration relies on the “*c* value”, defined as *c* = *nP*_t_/*K*_D_, where *P*_t_ is the concentration of protein in the cell, “*n*” is the number of binding sites per protein molecule, and *K*_D_ is the dissociation constant. To accurately estimate the affinity of binding at a particular site it is optimal to keep the *c* value between 20 and 100.^62^ However, it is important to point out that when attempting to capture multiple equilibria, it is often unlikely that the properties of sites with overlapping equilibria can be fully discerned from data fitting, as noted previously.^27^ Nonetheless, a semi-quantitative evaluation can still give meaningful insights.

For ESA, the acquired ITC data could be fitted to a 2-sequential-binding-sites model, yielding a primary binding site (log *K*_ITC_ = 4.98) and a secondary binding site (log *K*_ITC_ = 3.69) (Fit 1–1 in Table S3†). The data could also be fitted using a 3-sequential-sites model, again yielding a relatively high (log *K*_ITC_ = 5.20), medium (log *K*_ITC_ = 4.49; 5 times weaker than site 1) and low affinity site (log *K*_ITC_ = 3.36) (Fit 1–2 in Table S3†). Additionally, a 3-sets-of-sites model was also used to further examine possible stoichiometries of Co^2+^-binding to ESA (Fit 2–1 in Table S3†), where the data obtained from the 3-sequential-sites model were used but N3 (the stoichiometric constant corresponding to the weakest affinity site(s)) was fixed to 2 or 3, and the corresponding *K*_ITC_ and Δ*H* values for the weak site(s) were allowed to vary. This approach also yielded reasonable fits (Fit 3–1 in Table S3†) supporting the concept that multiple weak-affinity Co^2+^ binding sites may be present on ESA.

For the binding of Co^2+^ to HSA data, fitting of the data using a range of models suggested that there were at least three observable (sets of) cobalt binding sites on HSA under the chosen conditions. The simplest model that yielded a reasonable fit was a 3-sequential-sites model; Fit 1–2 in Table S3,† representing one site with relatively high affinity (log *K*_ITC_ = 5.65), one 6-times weaker site (log *K*_ITC_ = 4.86), and a low-affinity site (log *K*_ITC_ = 3.50). This is in broad agreement with previous studies, where at least three Co^2+^ sites in HSA have been identified, with log *K* values between 3.9–4.9.^22,26^ The presence of multiple lower-affinity Co^2+^-binding sites on HSA was then examined using a 3-sets-of-sites model as described above for ESA (Fit 2–2 in Table S3†), with parameters for the first two sites fixed to the values obtained in Fit 1–2. Again, the model could accommodate increased stoichiometries for the weakest set of sites (Fit 3–2 in Table S3†). These results support the concept that besides a primary and a secondary site, multiple weak-affinity Co^2+^ binding sites may be present on both HSA and ESA.

### 3.4. Co^2+^-binding properties of the primary and secondary sites of HSA

**Fig. 6:**
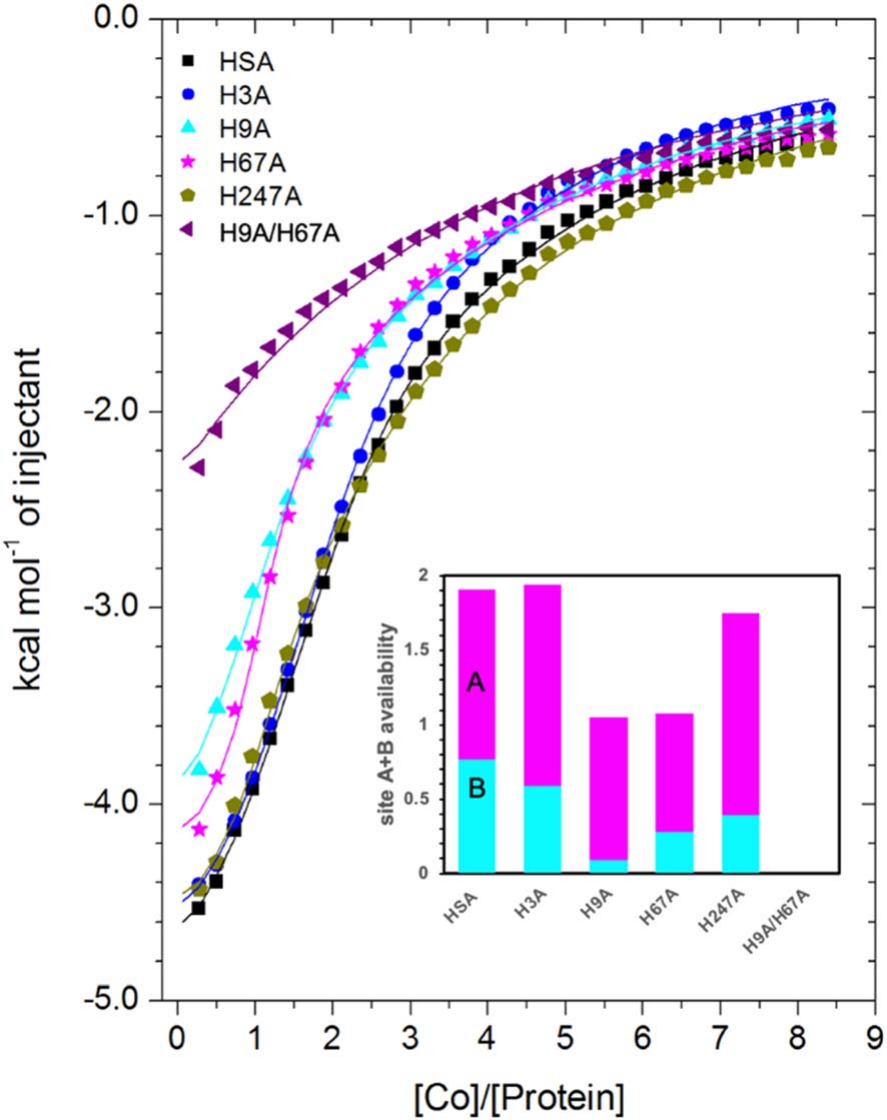
Measurement of Co^2+^ binding to plasma-purified HSA and different mutant HSA forms using ITC. In all cases, 50 μM of respective protein was titrated with 2 mM cobalt(ii) chloride solution for 35 injections. The data of HSA, H3A and H247A were fitted with a 3-sequential-sites model (Fits 1–2); the data of H9A and H67A were fitted with a 2-sequential-sites model (Fits 1–1); the data of H9A/H67A were fitted with a single-site model in MicroCal Origin 7.0 software (Fits 1–3, Table S3†). Inset: overall Co^2+^-binding capacity of sites A + B for HSA and different mutant forms of human albumin estimated by 3-sets-of-sites model (Fits 2–2). Of note, no error bars are shown, but estimated errors for the overall stoichiometry were at least ±0.2, based on the variation of *N* values for HSA between fitting using a 3-sequential-sites model (Fit 1–2; where *N* = 1) and after fitting using a 3-sets-of-sites model (Fits 2–2).

Site-directed mutagenesis was employed to investigate the involvement of specific residues implicated from crystallography (and other studies)^22,26,28^ in Co^2+^-binding. For this, H3A, H9A, H67A, H247A and H9A/H67A mutant forms of HSA were generated, and their ability to bind Co^2+^ was assessed using ITC (Fig. 6; raw data corresponding to titrations shown are provided in Fig. S10–S14;† note that His247 in HSA corresponds to His246 in ESA). The folded states of wild-type and mutant forms of HSA were assessed by ^1^H-NMR spectroscopy. Based on spectral dispersion and characteristic high-field shifted methyl proton signals, all HSA variants adopted a similar overall fold (Fig. S15†). Additionally, Co^2+^-binding capacity was compared between HSA and recombinant wild-type HSA (rHA) by ITC (Fig. S16 and Table S3;† raw data corresponding to titrations shown are provided in Fig. S9 and S17†). Using a 3-sequential-sites model, the result suggested that Co^2+^ binding to rHA was in broad agreement with that to HSA, yielding a relatively high (log *K*_ITC_ = 5.57), medium (log *K*_ITC_ = 4.89) and low affinity site (log *K*_ITC_ = 3.54; Table S3†). Hence, it was deemed appropriate to use the previously acquired data for HSA as control in the ITC studies of the mutant proteins.

Inspection of the ITC data plotted in Fig. 6 immediately indicates that the Co^2+^-binding capacity of H9A and H67A mutants is markedly reduced relative to HSA. Based on the expectation that removal of a critical histidine residue in a site would essentially remove this site, the 3-sequential-sites results previously obtained for HSA were used to fit the corresponding data obtained for each mutant using a 3-sets-of-sites model with log *K*_ITC_ and Δ*H* values for each site fixed and all three *N* values allowed to vary. This permitted a useful assessment of potential stoichiometric changes (*N* values) for each protein (Fit 2–2 in Table S3† and Fig. 6). Bearing in mind the considerations regarding *c* values as well as overlapping equilibria explained in Section 3.3, it will be most appropriate to consider the availability of sites 1 and 2 together for the following discussion.

Compellingly, the Co^2+^-binding capacity at the highest-affinity site (site 1) was most dramatically reduced in H9A, where N1 decreased to 0.1 (corresponding to site B in inset in Fig. 6 and Table S3†), suggesting that His9 is involved in coordinating Co^2+^ at the highest affinity site, proposed to be site B by Mothes and Faller.^22^ Crucially, a site involving His9 was present in both our Co^2+^–ESA and –HSA structures (Table 2), but also in a Zn^2+^–ESA and Zn^2+^–HSA structures.^19^ The chemical shifts of ^113^Cd^2+^ bound to site B in a range of mammalian serum albumins, including ESA and HSA, are around 24–28 ppm. This had suggested that Cd^2+^ co-ordinates at site B *via* a single N-ligand and multiple O-ligands.^63^ Together with these previous data, the studies of the H9A mutant here strongly suggest that His9 is this single N-ligand, with the octahedral coordination sphere completed by O-ligands from water molecules and one or more carboxylate(s). Asp13 was bound directly to the metal in either Zn^2+^ or Co^2+^ forms, whilst it appears that Asp254 (Asp255 in HSA) may bind in either inner- or outer sphere mode, depending on the metal ion. We note that N2 was also slightly reduced relative to the HSA data (corresponding to site A in bar chart in Fig. 6 and Table S3†), but attribute this to the inability of this direct titration approach to resolve overlapping equilibria, in accordance with our remarks in Section 3.3.

The Co^2+^-H67A ITC data indicated an apparent effect of this mutation on both equilibria for the primary and secondary sites, again due to overlapping equilibria and sub-optimal *c* values. Although we cannot exclude that the H67A mutation affects site 1 indirectly and/or *vice versa*, H67A is the only mutant for which a clear reduction in N2 (from 1.14 to 0.8, Table S3†) together with a reduction in the sum of N1+N2 (from 2.0 to 1.08, same as for the H9A mutant) was observed (bar chart in Fig. 6). Therefore, it is most likely that a site involving His67 corresponds to the second strongest Co^2+^ binding site on HSA. This is broadly consistent with the observations of Mothes and Faller, as well as of Sokolowska *et al.*, who suggested that site A is a secondary site for Co^2+^-binding.^22,26^

The involvement of His9 and His67 in the two strongest Co^2+^ binding sites is corroborated by the ITC results for the H9A/H67A double mutant, which displayed the greatest reduction in Co^2+^-binding capacity of the mutant HSA forms we examined (Fig. 6). Data fitting of Co^2+^ binding to H9A/H67A using a 3-sets-of-sites model suggested a reduction in both N1 and N2 to 0, with a value of 1.1 obtained for N3 (inset in Fig. 6 and Table S3†), suggesting that only binding at the weakest-affinity Co^2+^-binding site(s) remained after the removal of important residues engaged in site B (His9) and site A (His67).

The results obtained by ITC were further corroborated by circular dichroism (CD) experiments (Figures S18–S20, Table S4† and supplementary analysis of the CD data). These experiments were performed at a higher concentration of HSA (0.5 mM) titrating cobalt(ii) chloride from 1 to 10 molar equivalents. As shown previously by Sokołowska *et al.*,^26^ cobalt binding to HSA can be observed in the near-UV-visible range (wavelengths from 300 to 800 nm), giving 3 prominent bands with extrema at 325 nm (minimum), 420 nm (maximum) and 550 nm (minimum), respectively. The resulting spectra from the CD experiments show an average of the contribution of each occupied binding site. However, each binding site has a different signature in these spectra; for instance, it might be that binding at one site results in a negative band at 325 nm whilst all the other binding sites result in a positive band, giving ultimately a positive average. In addition, some binding sites might be CD-spectroscopically silent. Another caveat is the fact that sites A and B have micromolar affinities for Co^2+^ that cannot be quantified at the millimolar concentrations required for these CD experiments (0.5 to 5 mM). Therefore average affinities of the mutants H9A, H67A and the H9A/H67A double mutant for cobalt(ii) derived from the CD data will be dominated by binding to weaker sites, and are not expected to be drastically different from the WT. However, the contribution of the high-affinity binding sites might still impact the average spectra in a detectable manner, with mutant spectra and affinity trends different from the wild-type. To test this hypothesis, we extracted the CD values at each of the extrema and plotted the values against the cobalt(ii) concentration. The data were then fitted using a Hill's model which gave different Hill's constants, relating to the average affinity of the contributing binding sites at each wavelength (*K*_D_) (Table S4†). Comparing the binding curves of the mutants and the native protein taken at 325 nm, the mutants were very close to each other, and the *K*_D_ values were not significantly different, although a similar trend was seen at all wavelengths considered, whereby affinity values derived for H9A were always lower than those for H67A and native albumin, whilst the double mutant always displayed the lowest affinity. Following our hypothesis whereby affinities would rank B > A > other sites, it follows that *K*_D_ values should rank: H9A-H67A > H9A > H67A > WT, and indeed this was observed (Fig. S20† and supplementary analysis of the CD data give further details).

Collectively, this allows us to conclude that (i) Sites 1 and 2 correspond to site B and site A, respectively; (ii) loss of His9 or His67 reduces the overall site A + B availability from 2 to 1, with site A + B availability reduced to 0 in the His9/His67 double mutant; (iii) none of the other single mutants decreased site A + B availability to the same degree as the H9A or H67A mutant. Therefore, the obtained results suggest that sites B and A are critical Co^2+^-binding sites for HSA, with the resultant affinity constants implying that Co^2+^ binds site B more strongly than site A, in full agreement with previous suggestions based on spectroscopic and ITC evidence on wild-type HSA.^22,26^

### 3.5. Comparison of Co^2+^ and Zn^2+^-binding to site A

For Zn^2+^–HSA and Zn^2+^–ESA, the intact site A is formed by three protein ligands: His67 from domain I, and His247 (246 in ESA) and Asp249 (248 in ESA) from domain II. However, in the crystals soaked in high concentrations of Zn^2+^ (≥30 mM), an alternative conformation of the sidechain of His247 was observed together with the binding of a second Zn^2+^ ion to this residue only, with the original “site A” Zn^2+^ only bound to His67 and Asp249.^19^ His247 did also not participate in Co^2+^ binding in the ESA structure presented here, raising the question whether this binding mode is a consequence of crystal soaking or has relevance to solution conditions. To explore whether His247 was involved in high-affinity Co^2+^ binding in solution, the H247A mutant was examined. Although this mutation appeared to affect both primary and secondary sites, the sum of N1 + N2 was however still 1.75 (bar chart in Fig. 6); therefore, the role of this residue in Co^2+^ binding seems to be decisively less critical than that of His9 or His67. Whilst it cannot be excluded that absence of His247 could affect binding at the His9 site allosterically (*e.g.* by destabilisation of the domain I-domain II interface), its involvement in an “intact” site A makes a more direct effect on this site more likely. The fact that the impact of the H247A mutation appears to be less severe than the H67A mutation may indicate that the loss of interaction with His247 (if it occurs in solution) can be partially compensated: indeed, in the ESA–Co^2+^ structure when His246 is not part of the binding site, Glu251 can coordinate in outer sphere mode (Fig. S21†). On balance, we suggest that in solution, His247 might be an optional ligand in site A for Co^2+^, even though this is not reflected in the X-ray crystal structure.

### 3.6. NTS: a tertiary cobalt-binding site

Finally, examination of Co^2+^ binding to the H3A mutant by ITC revealed a slight change in overall stoichiometry (reduction from 3.0 to 2.7, Fig. S22†), with reductions focused on N3 (the weakest of the 3 sets of sites), with decreases from 1.1 in HSA to 0.8 (Table S3†). The proposed involvement of His3 in Co^2+^-binding has been controversial, with reduced binding at this site due to ischaemia-related modifications previously proposed to be the basis of the ACB assay.^24^ However, consistent with previous studies that have rejected this assumption,^26,27^ the results herein do not support involvement of this residue as part of a primary or secondary Co^2+^-binding site on HSA.

In plasma the majority of the 0.1–1.5 μg L^−1^ Co^2+^ is primarily bound to serum albumin, with 5-12% estimated to be free.^64^ Under normal physiological conditions, Co^2+^ would mainly be expected to bind HSA at site B. However, dramatic increases in cobalt concentrations have been reported in patients with hip or knee joint replacements made of cobalt alloys.^65^ In some cases, systemic cobalt levels (in collected serum) were above 200 μg L^−1^ (3.4 μM) and thus potentially orders of magnitude higher at the site of the implant.^66^ Under such conditions, the excess Co^2+^ ions could occupy weaker Co^2+^-binding sites, which could lead to pathological effects.

### 3.7. Effect of fatty acids on cobalt binding

HSA is the main circulatory transporter of NEFAs and there are at least seven NEFA binding sites (FA1-7) on HSA, three of which are considered high-affinity sites (FA2, FA4 and FA5).^59^ Among these sites, FA2 binds long-chain NEFAs with high affinity, and this interaction disrupts metal ion (*e.g.* Zn^2+^ and Cd^2+^) binding at site A.^32^ Significantly, the long-chain NEFA palmitate (C16 saturated NEFA), one of the most common physiological NEFAs found in the body, almost completely abrogated Zn^2+^ binding at site A when present at pathophysiological concentrations.^34^ NEFAs might also affect zinc binding at site B: In our previous study, an effect of myristate (C14 saturated NEFA) on Zn^2+^ binding affinity to BSA at site B was also observed,^32^ although this result is contradictory to subsequent observations for Zn^2+^–HSA binding, where site B was not significantly affected.^67^ To evaluate the influence of NEFAs on Co^2+^ binding to HSA at site A (His67 and His247 site) and site B (His9 site) in solution, titration of 2 mM cobalt chloride into 50 μM of wild-type and mutant HSAs (H9A, H67A and H247A) was carried out in the presence of 5 mol. eq. of palmitate (corresponding raw data of titrations shown are provided in Fig. S23–S26†).

**Fig. 7:**
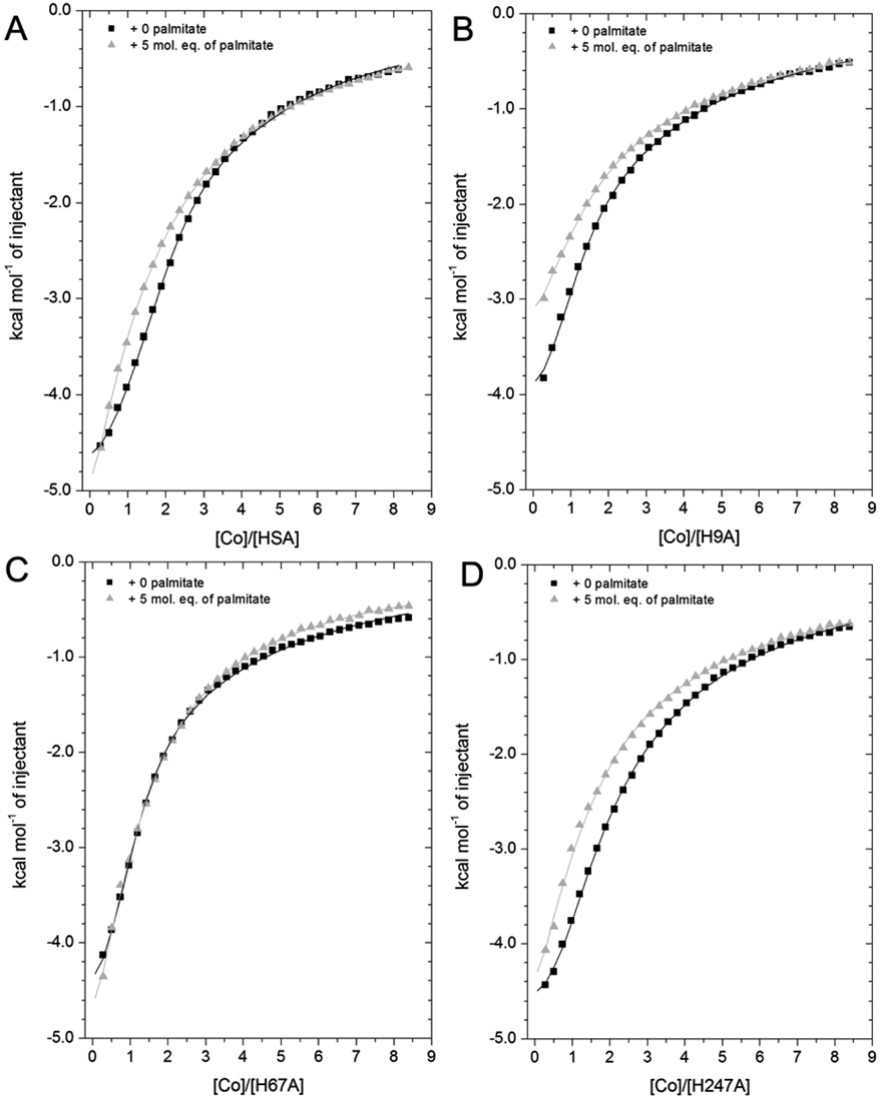
Competition between Co^2+^ and palmitate binding to (A) HSA and mutant HSAs (B, H9A; C, H67A; D, H247A) studied by ITC. In all cases, 2 mM Co(ii) chloride was titrated into 50 μM respective protein in the presence of 0 or 5 mol eq. of palmitate. (A) The data of HSA were fitted with a 3-sequential-sites model (in absence and presence of palmitate; Fit 1–2 in Table S3†); (B) the data of H9A (in absence and presence of palmitate) were fitted with a 2-sequential-sites model (Fit 1–1 in Table S3†); (C) the data of H67A (with or without 5 mol. eq. of palmitate) were also fitted with a 2-sequential-sites model (Fit 1–1 in Table S3†); (D) the data of H247A (in absence and presence of palmitate) were fitted with a 3-sequential-sites (Fit 1–2 in Table S3†) using MicroCal Origin 7.0 software.

As shown in Fig. 7A, the Co^2+^-binding capacity of HSA was compromised by the addition of 5 mol. eq. of palmitate, as previously observed for Co^2+^- binding to BSA.^27^ A 2-sequential-sites model (Fit 1–4 in Table S3†), based on the hypothesis that NEFA binding would completely remove site A (a model that appropriately describes the effect on Zn^2+^)^32,34^ did not yield a satisfactory fit. Crystallography and the H247A ITC data already suggested that there are marked differences between Zn^2+^ and Co^2+^ binding. Hence, we explored the hypothesis that NEFA binding might (i) not abolish binding at a site completely, and (ii) affect both sites A and B.

Indeed, a 3-sequential-sites model had a dramatically improved goodness-of-fit. Both log *K*_ITC_ values for sites B and site A decreased, by 1.22 and 0.87, respectively (Fig. 7 and Table S3†). This is consistent with the crystallographic data that (i) showed the possibility of Co^2+^ being retained in site A in a conformation that was closer to NEFA-bound ones than NEFA-free ones (Fig. 4), and (ii) suggested loss of outer-sphere coordination to Asp255 in site B.

As shown in Fig. 7B, the Co^2+^-binding capacity of H9A HSA was decreased by the presence of 5 mol. eq. of palmitate. In accordance with a 3-sequential sites model being appropriate to describe the wild-type data, the H9A data were modelled with 2-sequential-sites fits. Site A affinity (log *K*_ITC_ of the medium-affinity site) decreased from 4.85 to 4.47 (*i.e.* by 0.38). A 2-sequential-sites model was also applied to the H67A data, where site B affinity (log *K*_ITC_ of the high-affinity site) decreased from 4.85 to 4.27 (*i.e.* by 0.58). This would be consistent with the effects of NEFA binding on Asp254/255 in the outer coordination sphere of site B (Fig. 3B and S7†). This result suggests that susceptibility to NEFA binding largely involves site A, but that site B may also be impacted by NEFA binding.

Finally, the Co^2+^ binding affinity of the H247A mutant was also weakened by the addition of 5 mol. eq. of palmitate (Fig. 7D). Based on the observation that the H247A mutation does not remove a major binding site, 3-sequential-sites models were used to examine the effect of NEFA on sites A and B. Both log *K*_ITC_ values of high- and medium-affinity sites decreased, by 0.83 and 0.55, respectively. Alternative fits based on the hypothesis that site B was not affected by NEFA binding (Fits 1–4 (for HSA, H67A, and H247A + palmitate and Fit 1–3 for H9A + palmitate) in Table S3†) were also explored, but in each case the fits were unsatisfactory (large Chi^2^).

Summarily, our findings from ITC of wild-type and mutant HSAs in the presence of palmitate indicate that both sites A and B are affected by NEFA binding, consistent also with observations from crystallography. The trend observed for wild-type HSA was qualitatively in broad agreement with that made earlier for BSA.^27^ However, the firm identification of site B, the structural characterisation of both sites A and B when bound to Co^2+^, and the structural impact of NEFA binding on them, has now allowed for the first time to appreciate that it is more appropriate to regard the effect of NEFAs on Co^2+^ binding as a reduction in affinity rather than a complete loss of site availability as is valid for Zn^2+^.

The studies on human albumin here demonstrate that the mere addition of fatty acids to HSA compromises its Co^2+^-binding affinity, supporting our previous hypothesis that IMA corresponds to HSA with an increased number of NEFA molecules bound. Previously, IMA had been proposed to correspond to HSA with an impaired N-terminus displaying deficient Co^2+^-binding capacity, but this has never been demonstrated experimentally. In contrast, the structural changes elicited by NEFAs binding at site FA2 on both sites A and B provide a straightforward explanation of the observed decrease in Co^2+^ affinity that is characteristic of IMA, and is also consistent with the correlation between NEFA and IMA levels observed in the clinic.^68^ Our findings support the notion that also in HSA elevated NEFAs observed in myocardial ischemia and many other conditions play a critical role in decreased Co^2+^-binding to IMA^28^ rather than N-terminal modification.

## 4. Conclusions

In conclusion, we have identified and structurally characterised Co^2+^–ESA and HSA binding sites by X-ray crystallography and biochemically characterised Co^2+^ binding to ESA and HSA by ITC, including mutagenesis for the latter. Our results here are in firm agreement with previous data that suggest that Co^2+^ preferentially binds to site B, followed by site A. Furthermore, 5 mol. eq. of palmitate can efficiently decrease Co^2+^ binding to HSA, in broad accordance with previous results for BSA.^27,34^ All three (sets of) sites studied here bear clinical importance. We have conclusively identified site B as the primary Co^2+^ binding site in the circulatory system; this is of relevance not only for low-grade cobalt-toxicity, but also underpinning a more accurate molecular understanding of the ACB test, where previous hypotheses focused almost exclusively on site A. Indeed, elevated NEFA levels impacted Co^2+^ binding to site A of HSA, but also to site B, as shown by both crystallography and ITC. Finally, the multiple weak-affinity Co^2+^-binding sites, suggested by our X-ray structure and supported by ITC analysis, may be of relevance to autoimmune conditions associated with Co^2+^-toxicity, especially in the context of MoM implants.

A major insight gained from the present work is that the binding modes of Zn^2+^ and Co^2+^ in both sites A and B differ more substantially than previously appreciated, with outer-sphere coordination playing a more important role in the binding of Co^2+^. Varying the myristate (C14:0) and cobalt(ii) concentrations when generating crystals allowed us to access different snapshots of the binding sites. These provided insight into the fatty acid-mediated structural changes that diminish the affinity of HSA toward Co^2+^. This highlights the fact that applications of the ACB assay, which often extend beyond myocardial ischemia, should be assessed cautiously and in the context of conditions with elevated NEFA levels. Finally, HSA is also a critical carrier of other metal ions, drugs, hormones and NEFAs; therefore, the details of Co^2+^ binding locations and the respective binding properties at each site allow further understanding of potential allosteric mechanisms regulating ligand binding. Our recent study suggests that such mechanisms can differ between albumins from different mammals,^69^ so characterisation of these sites on albumins from both equine and human species is important.

## Data availability

The atomic coordinates of the presented structures were deposited in the RCSB PDB with accession codes 8EW4, 8EW7, 8EY5 for HSA and 7MBL for ESA. The ITC and CD data can be accessed at DOI: 10.17630/34d5ba8e-569d-4630-aac7-8c61a64928a0.

## Author contributions

D. W., M. G., M. P. C., D. R. C., I. G. S., S. A., U. S. L., and performed experiments (D. W. expressed and purified recombinant proteins; M. G., M. P. C., D. R. C. and I. G. S. carried out X-ray crystallography; D. W. and U. S. L. Performed NMR experiments; D. W. and S. A. performed ITC experiments; D. W. and R. F. carried out circular dichroism experiments); D. W., M. G., D. R. C., M. P. C., I. G. S., R. F., U. S. L., C. A. B., W. M. and A. J. S. analysed and interpreted the results; D. W., M. G., D. R. C., M. P. C., I. G. S., U. S. L., C. A. B., W. M. and A. J. S. wrote the paper; U. S. L., C. A. B., W. M. and A. J. S. designed the research.

## Conflicts of interest

One of the authors (W. M.) notes that he has also been involved in the development of state-of-the-art software, data management and mining tools; some of these have been commercialized by HKL Research and are mentioned in the paper. W. M. is the co-founder of HKL Research and a member of the board. The authors have no other relevant affiliations or financial involvement with any organisation or entity with a financial interest in or financial conflict with the subject matter or materials discussed in the manuscript apart from those disclosed.

## Supplementary Material

SC-014-D3SC01723K-s001
