# Supplementary material for: Structural and biochemical characterisation of Co2+-binding sites on serum albumins and their interplay with fatty acids
Source: Chem Sci. 2023 May 23;14(23):6244–58. doi: 10.1039/d3sc01723k (PMC10266443; doi:10.1039/d3sc01723k)
Supplement: SC-014-D3SC01723K-s001 [file SC-014-D3SC01723K-s001.pdf]

Electronic Supplementary Material (ESI) for Chemical Science.
This journal is © The Royal Society of Chemistry 2023

Supplementary Information

Identification and structural characterisation of Co^{2+} binding sites on serum albumin

Dongmei Wu, Michal Gucwa, Mateusz P. Czub, David R. Cooper, Ivan G. Shabalin, Remi Fritzen, Swati Arya, Ulrich Schwarz-Linek, Claudia A. Blindauer, Wladek Minor, Alan J. Stewart.

**Table S1.**	**Fitting approaches used for ITC experiments.**
**Table S2.**	**Summary of myristic acid binding to HSA structures.**
**Table S3.**	**Fitting results for ITC experiments.**
**Table S4.**	**Fitting parameters for CD titrations.**
**Table S5.**	**Comparisons of the relative distance between OD2 of Asp254/5 and either NE2 of His9, OD2 of Asp13 or 1 of 7MBL, 8EW7, 8EW4 and 8EY5.**
**Figure S1.**	**Comparison of serum albumin structures.**
**Figure S2.**	**Structure of HSA (8EW4) in complex with myristate.**
**Figure S3.**	**Myristate binding at FA1.**
**Figure S4.**	**Comparison of different albumin structures.**
**Figure S5.**	**Structural comparison of the FA2 sites of 1BJ5 (in blue) and 8EW4 (in yellow).**
**Figure S6.**	**Impact of symmetry mate sidechains on sites A and B.**
**Figure S7.**	**Structural comparisons of Co^{2+} binding at site B or B'.**
**Figure S8.**	**ITC raw data. Full ITC data (including raw data) for Co^{2+} binding to 50 µM ESA.**
**Figure S9.**	**ITC raw data. Full ITC data (including raw data) for Co^{2+} binding to 50 µM HSA.**
**Figure S10.**	**ITC raw data. Full ITC data (including raw data) for Co^{2+} binding to 50 µM H3A human albumin.**
**Figure S11.**	**ITC raw data. Full ITC data (including raw data) for Co^{2+} binding to 50 µM H9A human albumin.**
**Figure S12.**	**ITC raw data. Full ITC data (including raw data) for Co^{2+} binding to 50 µM H67A human albumin.**
**Figure S13.**	**ITC raw data. Full ITC data (including raw data) for Co^{2+} binding to 50 µM H247A human albumin.**
**Figure S14.**	**ITC raw data. Full ITC data (including raw data) for Co^{2+} binding to 50 µM H9A/H67A human albumin.**
**Figure S15.**	**Assessment of protein structure in HSA and recombinant HSAs by 1H-NMR.**
**Figure S16.**	**Measurement of Co^{2+} binding to plasma-purified HSA and recombinant wild-type HSA using ITC.**
**Figure S17.**	**ITC raw data. Full ITC data (including raw data) for Co^{2+} binding to 50 µM recombinant wild-type HSA.**

Supplementary discussion of the circular dichroism data.

Figure S18. **Baseline corrected circular dichroism spectra obtained by titration of Co^{2+} with different HSAs ([HSA] 0.5mM, [Co^{2+}] 0.5mM to 5mM).**

Figure S19. **CD spectra obtained by subtraction of the (A) H9A, (B) H67A and (C) H9A/H67A spectra from the spectrum of HSA.**

Figure S20. **Plotted maxima for the three observed peaks at: (A) 325nm, (B) 420nm and (C) 550nm.**

Figure S21. **Co^{2+} binding at site A of 7MBL.**

Figure S22. **Overall Co^{2+}-binding capacity for HSA and mutant forms of human albumin.**

Figure S23. **ITC raw data. Full ITC data (including raw data) for Co^{2+} binding to 50 µM HSA in the presence of 5 mol. eq. of palmitate.**

Figure S24. **ITC raw data. Full ITC data (including raw data) for Co^{2+} binding to 50 µM H9A human albumin in the presence of 5 mol. eq. of palmitate.**

Figure S25. **ITC raw data. Full ITC data (including raw data) for Co^{2+} binding to 50 µM H67A human albumin in the presence of 5 mol. eq. of palmitate.**

Figure S26. **ITC raw data. Full ITC data (including raw data) for Co^{2+} binding to 50 µM H247A human albumin in the presence of 5 mol. eq. of palmitate.**

Table S1. Fitting approaches used for ITC experiments.

ITC fitting approaches for titration of Co^{2+} into ESA, HSA and mutant forms of human albumin (in absence or presence of 5 mol. eq. of palmitate). The values of K_{ITC} and ΔH in fit 2 and 3 were derived from fit 1. All entries labelled "v" were varied. Fitting results obtained based on these models are provided in Table S2.

Fit	Protein	Model	Fixed Parameters								
			High-affinity site			Medium-affinity site			Weak-affinity site(s)		
				log K_{ITC}	ΔH (cal/mol)		log K_{ITC}	ΔH (cal/mol)		log K_{ITC}	ΔH (cal/mol)
Fit 1-1		2-sequential binding sites		V	V		V	V			
Fit 1-2		3-sequential binding sites		V	V		V	V		V	V
Fit 1-3		1-sequential binding site		V	V						
Fit 1-4		2-sequential binding sites		4.50	V		V	V			
			N1	log K_{ITC}	ΔH (cal/mol)	N2	log K_{ITC}	ΔH (cal/mol)	N3	log K_{ITC}	ΔH (cal/mol)
Fit 2-1	ESA	3 sets-of-sites	V	1.59	-4912	V	3.10	-2579	V	2.29	-8191
Fit 3-1	ESA	3 sets-of-sites	V	1.59	-4912	V	3.10	-2579	2 or 3	V	V
Fit 2-2	HSA	3 sets-of-sites	V	4.50	-4821	V	7.30	-3934	V	3.14	-14690
Fit 3-2	HSA	3 sets-of-sites	V	4.50	-4821	V	7.30	-3934	2 or 3	V	V

Table S2. Summary of myristic acid binding to HSA structures. Sites FA1-7 were identified based on the locations of these described by Bhattacharya et al (doi: 10.1006/jmbi.2000.4158). In some structures, two myristate molecules were found to be present in FA7, these were labelled a and b. We identified fatty acids at two other locations which we named Pos8 and 9. The locations of all sites are shown in Figure S2. In the table '+' indicates that the site is occupied by a myristic acid molecule supported by electron density in the 2fo-fc map and '-' indicates that there was no electron density in 2fo-fc to support placement of ligand in this position.

Fatty acid-binding site	8EW4	8EW7	8EY5	UNPUB
FA1	+	-	+	+
FA2	+	-	-	-
FA3	+	+	+	+
FA4	+	+	+	+
FA5	+	+	+	+
FA6	+	-	+	+
FA7(a)	+	-	+	+
FA7(b)	+	-	+	+
Pos8	+	-	+	+
Pos9	+	-	+	+

Table S3. Fitting results for ITC experiments.

Fitting results for ITC experiments of titration of Co^{2+} into ESA, HSA, rHA and mutant forms of human albumin (in absence or presence of 5 mol. eq. of palmitate). '*' indicates that the corresponding parameter was fixed.

		High-affinity site		Medium-affinity site		Weak-affinity site(s)		
Fit	Protein	log K_{ITC}	ΔH (cal/mol)	log K_{ITC}	ΔH (cal/mol)	log K_{ITC}	ΔH (cal/mol)	Chi2/degree of freedom
Fit 1-1	ESA	4.98	-5205	3.69	-8540			1499
Fit 1-2	ESA	5.17	-4912	4.49	-2579	3.35	-8191	339.2
Fit 1-2	HSA	5.65	-4821	4.86	-3934	3.49	-14690	671.1
Fit 1-2	rHA	5.57	-4364	4.88	-2560	3.54	-9254	508.5
Fit 1-2	H3A	5.65	-4708	4.82	-4020	3.60	-10240	704.4
Fit 1-1	H9A			4.85	-4958	3.45	-15820	726
Fit 1-1	H67A	4.85	-5568			3.33	-18250	643.5
Fit 1-2	H247A	5.10	-5210	4.09	-7842	3.23	-14040	550.9
Fit 1-3	H9A/H67A					3.45	-20200	2674
Fit 1-4	HSA + palmitate	5.65*	-4173			3.65	-17490	5959
Fit 1-3	H9A + palmitate					3.61	-16360	970.8
Fit 1-4	H67A + palmitate	5.65*	-3917			3.65	-12380	8495
Fit 1-4	H247A + palmitate	5.65*	-3733			3.47	-18410	7057
Fit 1-2	HSA + palmitate	4.43	-8545	3.98	-2632	3.27	-13980	77.52
Fit 1-1	H9A + palmitate			4.47	-5169	3.26	-16520	236.8
Fit 1-1	H67A + palmitate	4.27	-9620			3.06	-13850	487.8
Fit 1-2	H247A + palmitate	4.27	-8926	3.54	-6075	1.18	-11720	1339

		N1	log K_{ITC}	ΔH (cal/mol)	N2	log K_{ITC}	ΔH (cal/mol)	N3	log K_{ITC}	ΔH (cal/mol)	Global goodness of fit
Fit 2-1	ESA	0.90	5.17*	-4912*	0.97	4.49*	-2579*	1.15	3.35*	-8191*	63.2%
Fit 3-1	ESA	0.93	5.17*	-4912*	1.03	4.49*	-2579*	2*	3.29	-4980	67.5%
Fit 3-1	ESA	0.97	5.17*	-4912*	0.99	4.49*	-2579*	3*	3.33	-3150	67.2%
Fit 2-2	HSA	0.77	5.65*	-4821*	1.14	4.86*	-3934*	1.06	3.49*	-14690*	43.7%
Fit 3-2	HSA	0.84	5.65*	-4821*	1.17	4.86*	-3934*	2*	3.54	-7240	44.2%
Fit 3-2	HSA	0.93	5.65*	-4821*	1.18	4.86*	-3934*	3*	3.59	-4470	45.7%
Fit 2-2	H3A	0.59	5.65*	-4821*	1.35	4.86*	-3934*	0.76	3.49*	-14690*	49.6%
Fit 2-2	H9A	0.09	5.65*	-4821*	0.96	4.86*	-3934*	1.07	3.49*	-14690*	30.5%
Fit 2-2	H67A	0.28	5.65*	-4821*	0.80	4.86*	-3934*	1.04	3.49*	-14690*	34.3%
Fit 2-2	H247A	0.39	5.65*	-4821*	1.36	4.86*	-3934*	1.14	3.49*	-14690*	57.6%
Fit 2-2	H9A/H67A	0	5.65*	-4821*	0	4.86*	-3934*	1.11	3.49*	-14690*	13.2%

Table S4. Fitted parameters of CD titrations. Parameters are the result of Hill's model fitting of the CD data.

Peak	Protein	a / Δθ		b / Δθ		Kd / mM		n	
		Value	SE	Value	SE	Value	SE	Value	SE
1	HSA	-0.19	0.42	13.07	1.24	2.17	0.17	2.67	0.49
	H9A	0.07	0.10	11.75	1.16	3.52	0.44	1.56	0.12
	H67A	0.28	0.23	7.04	0.58	2.12	0.13	3.08	0.56
	H9A/H67A	0.08	0.21	13.28	3.19	3.94	1.12	1.63	0.28
2	HSA	-0.07	0.31	8.27	1.64	2.44	0.54	1.80	0.46
	H9A	0.17	0.14	11.13	0.94	3.03	0.22	2.37	0.24
	H67A	-0.04	0.08	5.43	0.32	2.66	0.12	2.99	0.30
	H9A/H67A	0.04	0.07	19.28	6.52	10.00	4.57	1.18	0.11
3	HSA	0.01	0.17	5.70	0.64	2.23	0.23	2.26	0.42
	H9A	0.07	0.10	11.75	1.16	3.52	0.44	1.56	0.12
	H67A	0.05	0.08	7.66	0.65	2.97	0.33	1.51	0.12
	H9A/H67A	0.01	0.05	24.02	12.77	26.12	23.66	0.85	0.07

Table S5. Comparisons of the relative distance between OD2 of Asp254/5 and either NE2 of His9, OD2 of Asp13 or 1 of 7MBL, 8EW7, 8EW4 and 8EY5.

Distances were measured using Pymol.

	Corresponding Asp254/5
His9 in 7MBL	5.0 Å
His9 in 8EW7	7.5 Å
His9 in 8EY5	9.0 Å
His9 in 8EW4	9.6 Å
Asp13 in 7MBL	5.5 Å
Asp13 in 8EW7	8.6 Å
Asp13 in 8EY5	9.8 Å
Asp1 in 8EW4	11.8 Å

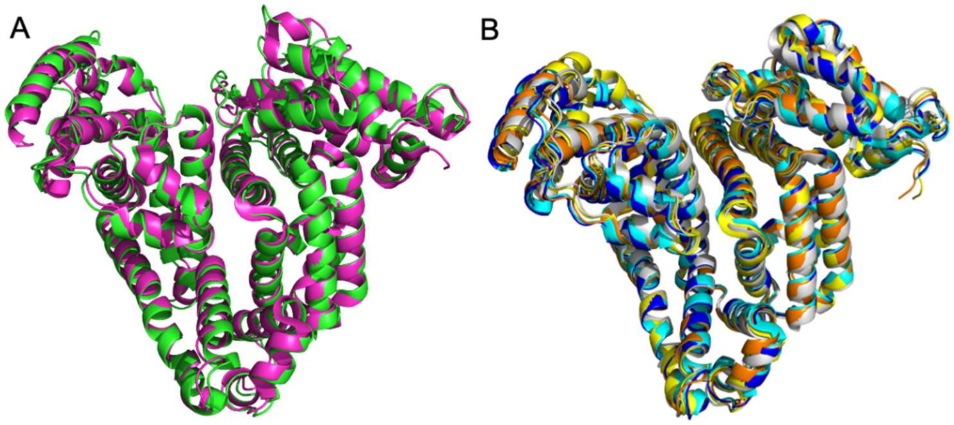

Figure S1. Comparison of serum albumin structures. A) Overlay of human and equine albumin structures without bound fatty acid. 1BM0 (unliganded HSA) is shown in green and 7MBL (Co^{2+}-ESA) is shown in magenta; fit for Cα RMSD=0.717 Å. B) Overlay of human albumin structures with bound fatty acids, fits of Cα relative to 8EW4 are shown. 8EW4 in yellow, 8EY5 in orange (RMSD=0.547 Å), 8EW7 in white (RMSD=0.847 Å), 1BJ5 (HSA with bound myristate) in blue (RMSD=0.908 Å), 1E7H (HSA with bound palmitate) in cyan (RMSD=1.106 Å).

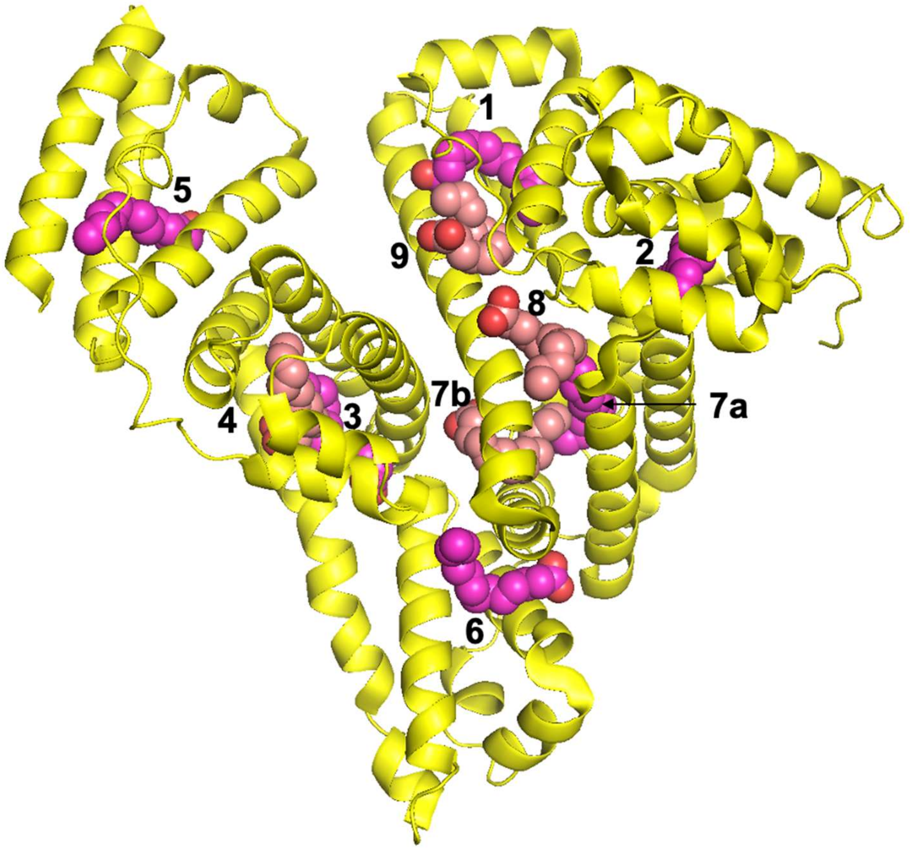

Figure S2. Structure of HSA (8EW4) in complex with myristic acid. The yellow ribbon represents the main protein chain. Locations of fatty acid binding sites are numbered. Carbon atoms are shown in salmon or magenta (two colors were chosen to provide better contrast where two myristate molecules bind in very close proximity); oxygen in red).

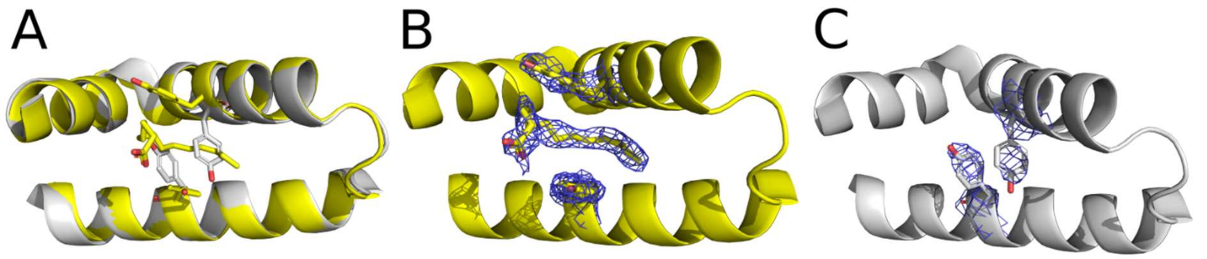

Figure S3. Myristate binding at FA1. A) Superposition of FA1 in HSA structures 8EW4 and 8EW7. 8EW4 is shown in yellow, and 8EW7 which possessed a lower number of bound myristate molecules is shown in white; fit for Cα of residues 119-170 RMSD=0.447 Å, only Tyr138 and Tyr161 are shown in stick representation. B) 8EW4 with electron density of 2fo-fc map around tyrosines and the modelled myristate. C) 8EW7 with electron density of 2fo-fc map around tyrosines.

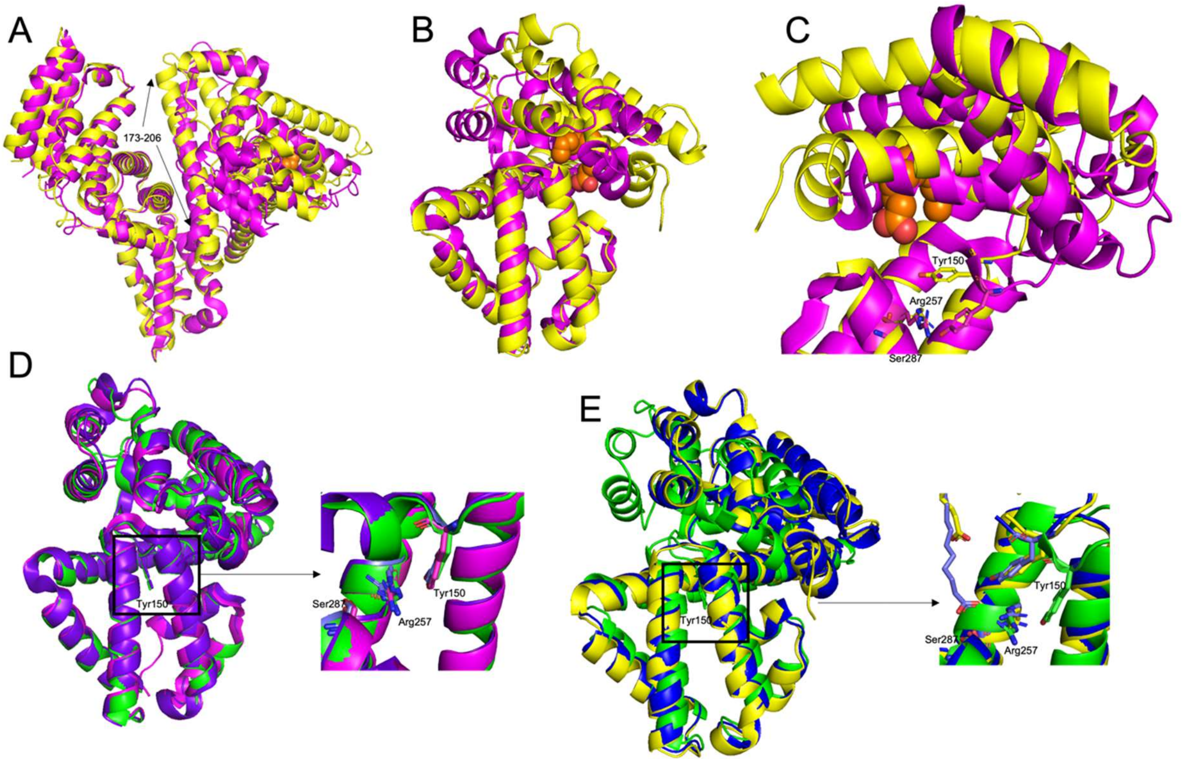

Figure S4. Comparison of different albumin structures. A) Global superimposition of 7MBL in magenta (ESA, no myristate bound) and 8EW4 in yellow (HSA, myristate bound). Myristate within the FA2 site is shown by orange spheres (RMSD=2.477 Å). The alpha-helix located between residues 173-206 are indicated by arrows. B) Superimposition where only residues 1-290 of 7MBL and 8EW4 are shown. C) Zoomed in image showing the FA2 sites. residues Tyr150, Arg257 and Ser287 are shown in stick representation. D) Superimposition of residues 1-290 and FA2 sites of 7MBL, 4F5T (in purple; unliganded ESA; RMSD=0.940 Å) and 1BM0 (in green; unliganded HSA; RMSD=0.781 Å). This indicates that cobalt(II) binding and species discrepancy does not influence the structure at FA2 sites, indicated by the identical positions of these three residues across these structures. E) Superimposition of residues 1-290 and FA2 sites of 1BJ5 (in blue; HSA with myristate bound),1BM0 (RMSD=3.241 Å) and 8EW4 (in yellow; HSA with myristate and cobalt bound; RMSD=1.158 Å). Myristate molecules are shown in stick representation with carbon shown in yellow or blue and oxygen shown in red. This suggests that conformational switch (domain IA relative to domain IIA) is caused by fatty acid binding, leading to the movement of Tyr150 (6.9 Å) towards the myristate molecule. Distance was measured using Pymol.

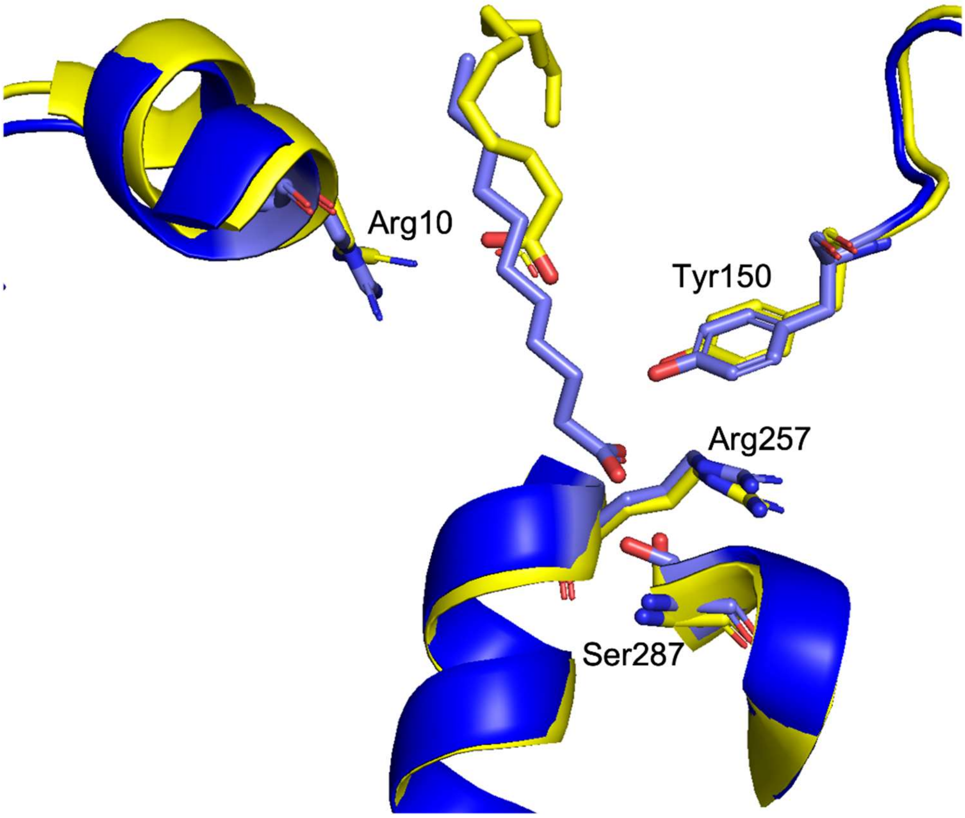

Figure S5. Structural comparison of the FA2 sites of 1BJ5 (in blue) and 8EW4 (in yellow). Myristate, Arg10, Tyr150, Arg257 and Ser287 are shown in stick representation and the amino acids are labelled. The myristate molecule bound in 8EW4 is located 6.4 Å from its position in 1BJ5, such that the carboxylic group of myristic acid associates with Arg10 rather than with Tyr150, Arg257 and Ser287. Distance was measured using Pymol.

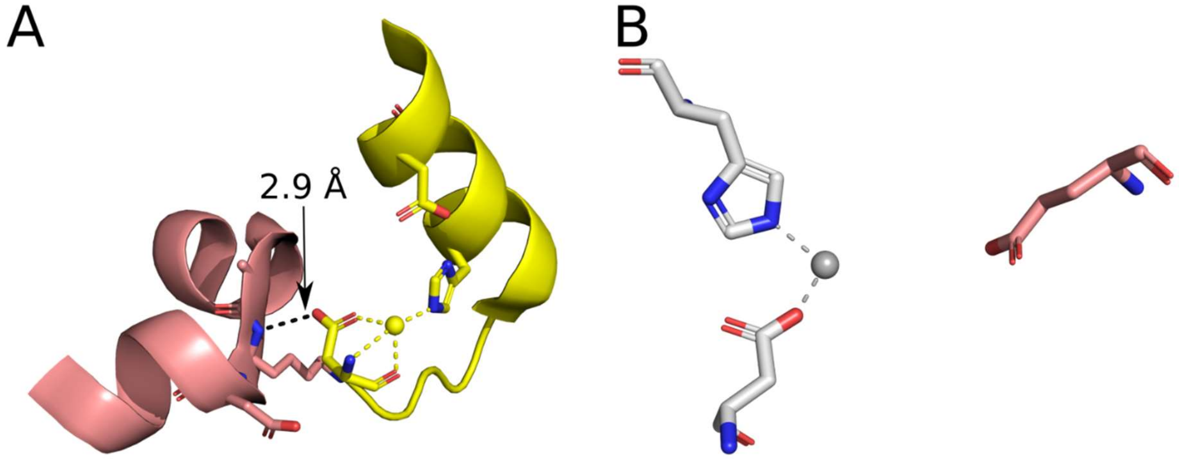

**Figure S6. Impact of symmetry mate sidechains on sites A and B`.** A) Site B` in 8EW4 structure in yellow, and an interaction from another chain in the crystal lattice shown in pink. A hydrogen bond of 2.9 Å is shown by the black arrow. The cobalt ion is shown as a yellow sphere. B) Site A (involving His67 and Asp249) in 8EW7 in white is shown. The neighboring residue Glu227 from another chain in the crystal lattice is shown in pink. The cobalt ion is shown as a white sphere.

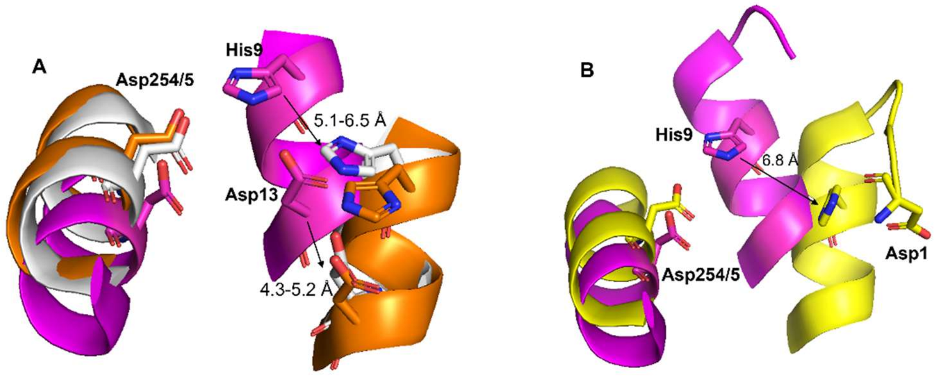

Figure S7. Structural comparisons of Co^{2+} binding at sites B or B'. A) Comparison of sites B in 7MBL (in magenta), 8EW7 (in white) and 8EY5 (in orange). In the myristate-bound structures, His9 and Asp13 move by 5.1-6.5 Å and 4.3-5.2 Å, respectively relative to their positions in 7MBL. B) Comparison of site B' between 8EW4 (in yellow) and 7MBL. The position of His9 differs by 6.8 Å between the structures. However, a difference in position of Asp1 cannot be measured as 7MBL lacks this residue.

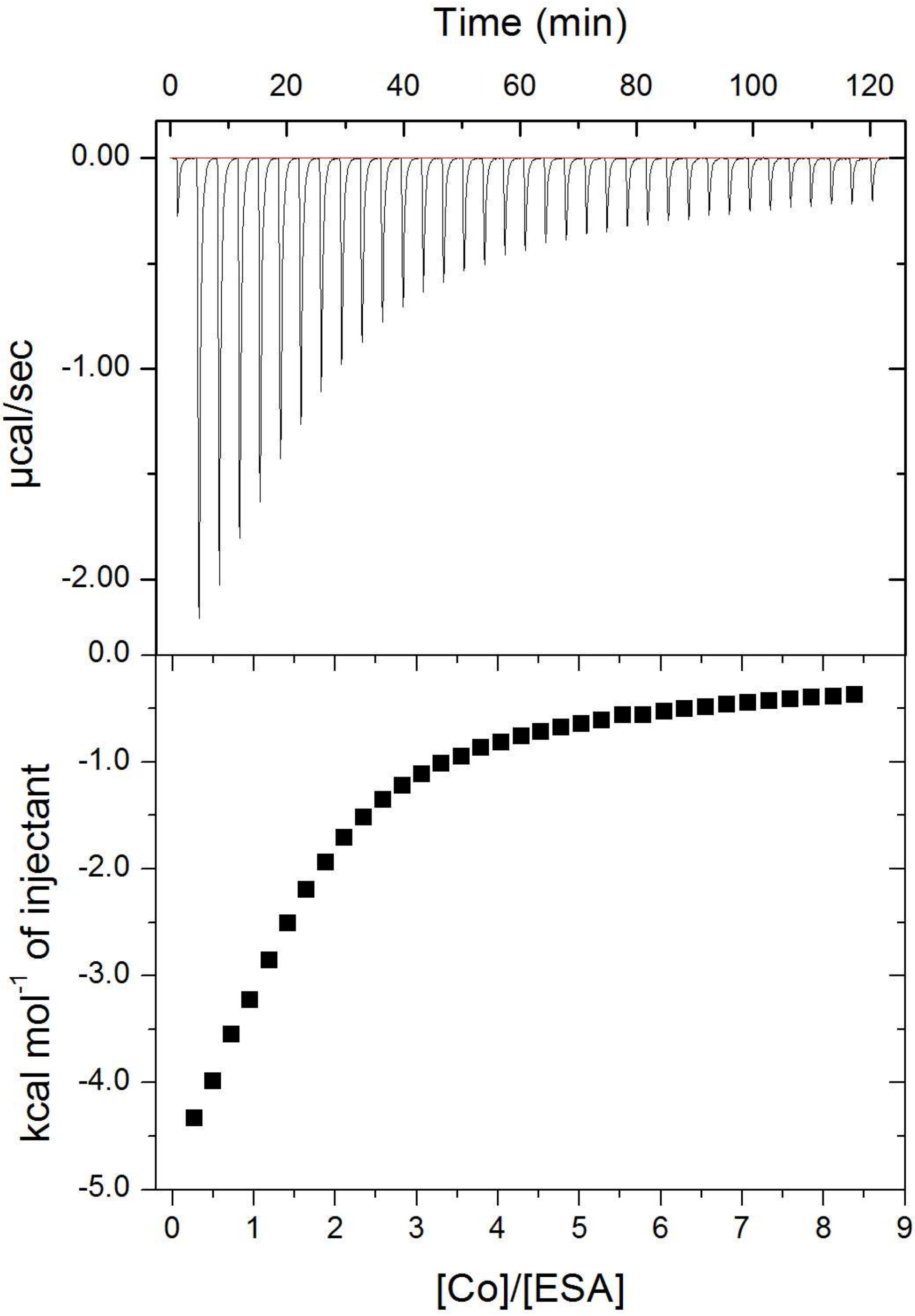

Figure S8. ITC raw data for Co^{2+} (2 mM) titration into ESA (50 µM). Upper plots: thermograms (thermal power required to maintain a zero-temperature difference between reference and sample cells in the calorimeter) showing the characteristic sequence of peaks corresponding to each ligand injection. Of note, the first titration was removed. Lower plots: binding isotherm showing normalized heats per peak as a function of molar ratio (ligand/protein) in the sample cell. Of note, the first titration point was removed for this plot. Corresponding data are shown in Figure 5.

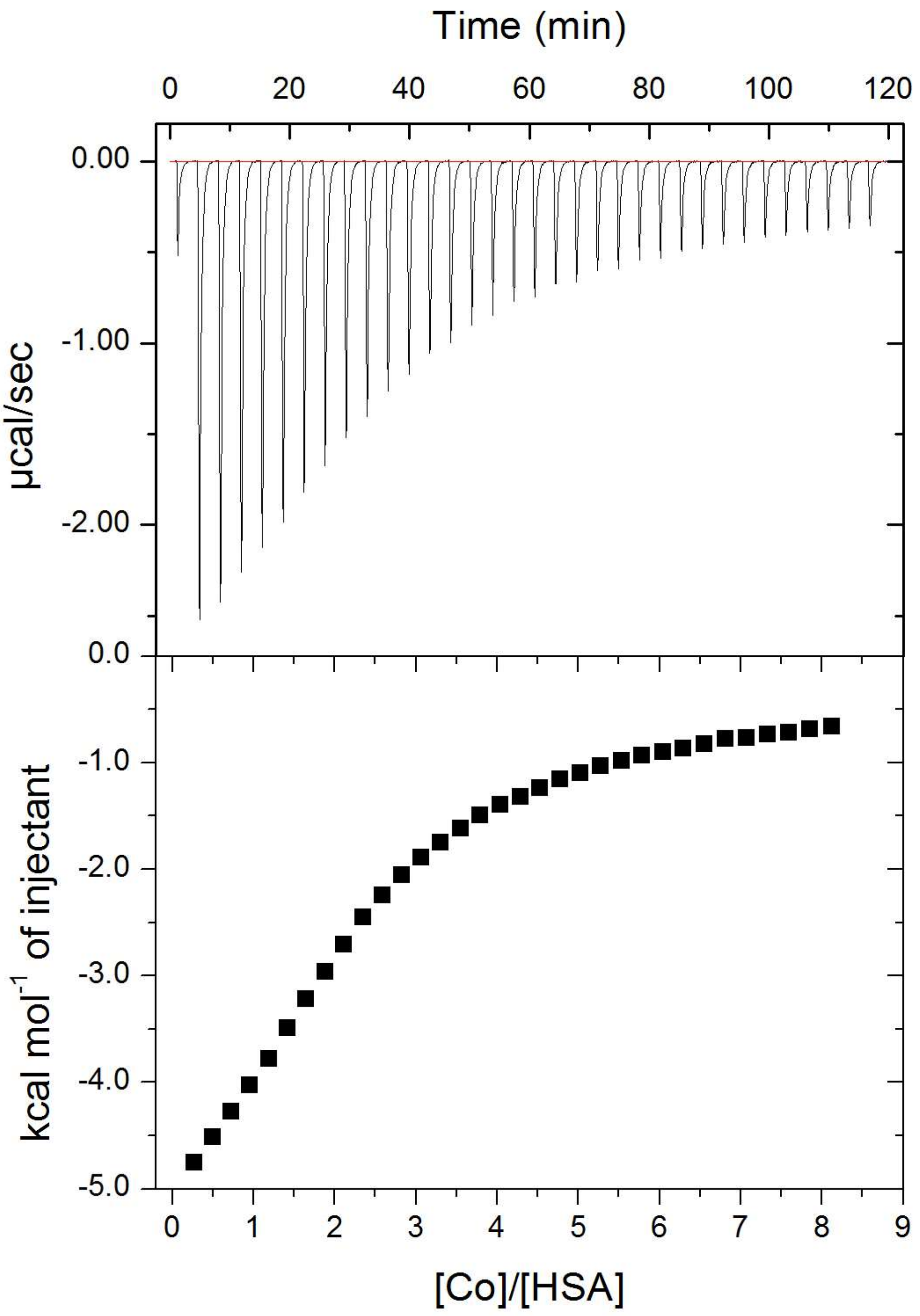

Figure S9. ITC raw data for Co^{2+} (2 mM) titration into HSA (50 µM). Upper plots: thermograms (thermal power required to maintain a zero-temperature difference between reference and sample cells in the calorimeter) showing the characteristic sequence of peaks corresponding to each ligand injection. Lower plots: binding isotherm showing normalized heats per peak as a function of molar ratio (ligand/protein) in the sample cell. Of note, the first titration point was removed for this plot. Corresponding data are shown in Figures 5, 6, 7A and S14.

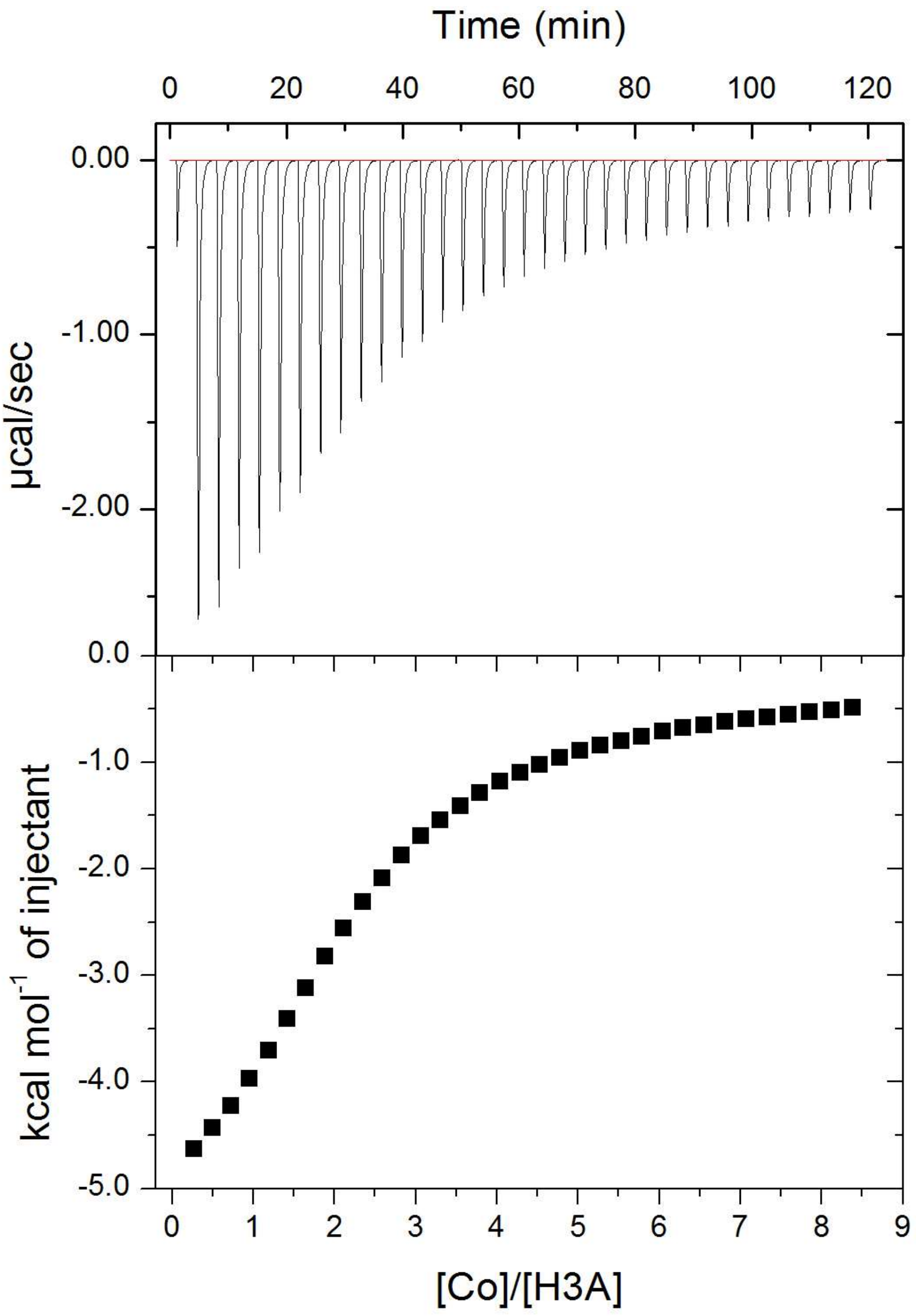

Figure S10. ITC raw data for Co^{2+} (2 mM) titration into H3A human albumin (50 µM). Upper plots: thermograms (thermal power required to maintain a zero-temperature difference between reference and sample cells in the calorimeter) showing the characteristic sequence of peaks corresponding to each ligand injection. Of note, the first titration was removed. Lower plots: binding isotherm showing normalized heats per peak as a function of molar ratio (ligand/protein) in the sample cell. Of note, the first titration point was removed for this plot. Corresponding data are shown in Figure 6.

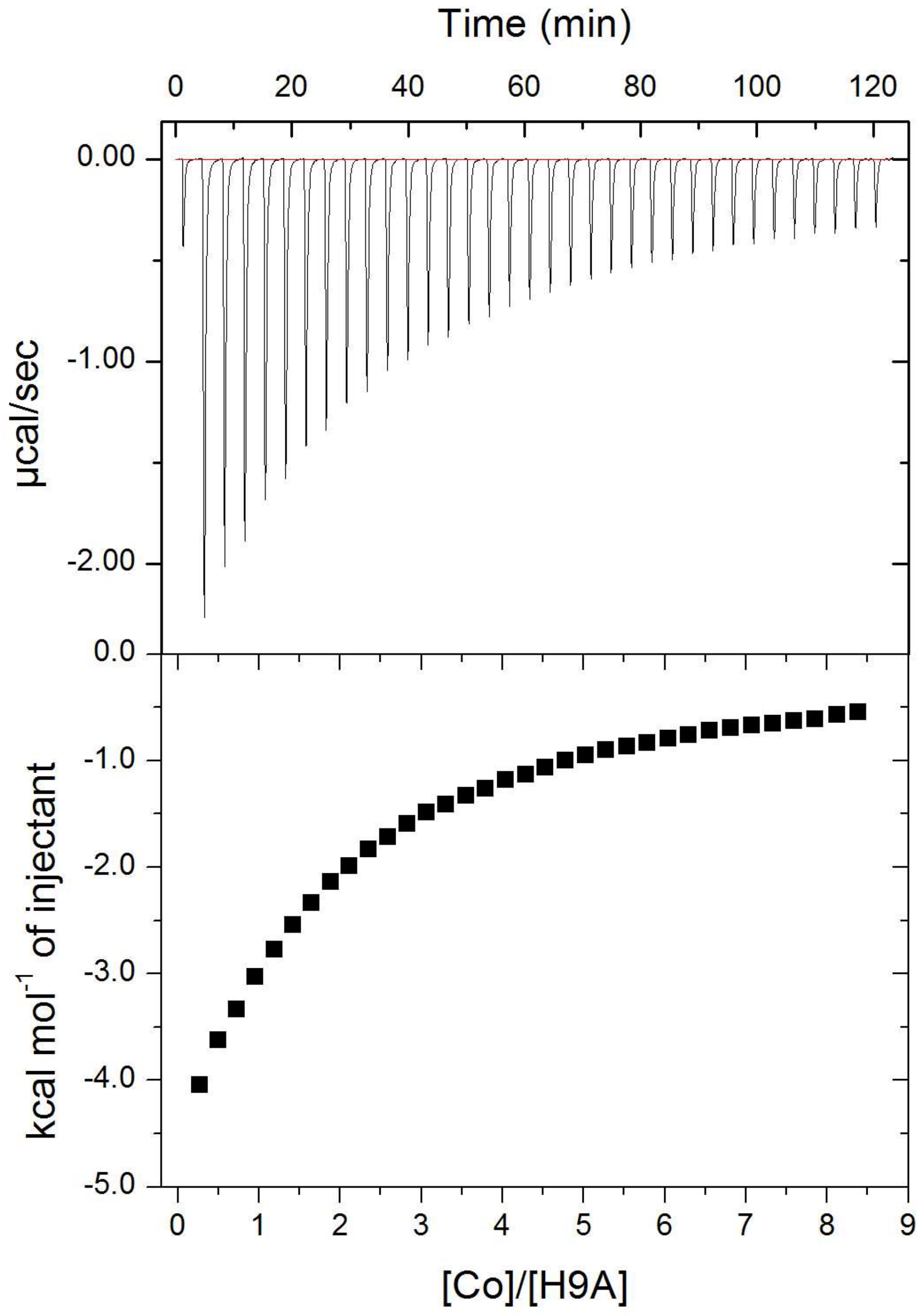

Figure S11. ITC raw data for Co^{2+} (2 mM) titration into H9A human albumin (50 µM). Upper plots: thermograms (thermal power required to maintain a zero-temperature difference between reference and sample cells in the calorimeter) showing the characteristic sequence of peaks corresponding to each ligand injection. Of note, the first titration was removed. Lower plots: binding isotherm showing normalized heats per peak as a function of molar ratio (ligand/protein) in the sample cell. Of note, the first titration point was removed for this plot. Corresponding data are shown in Figure 6 and 7B.

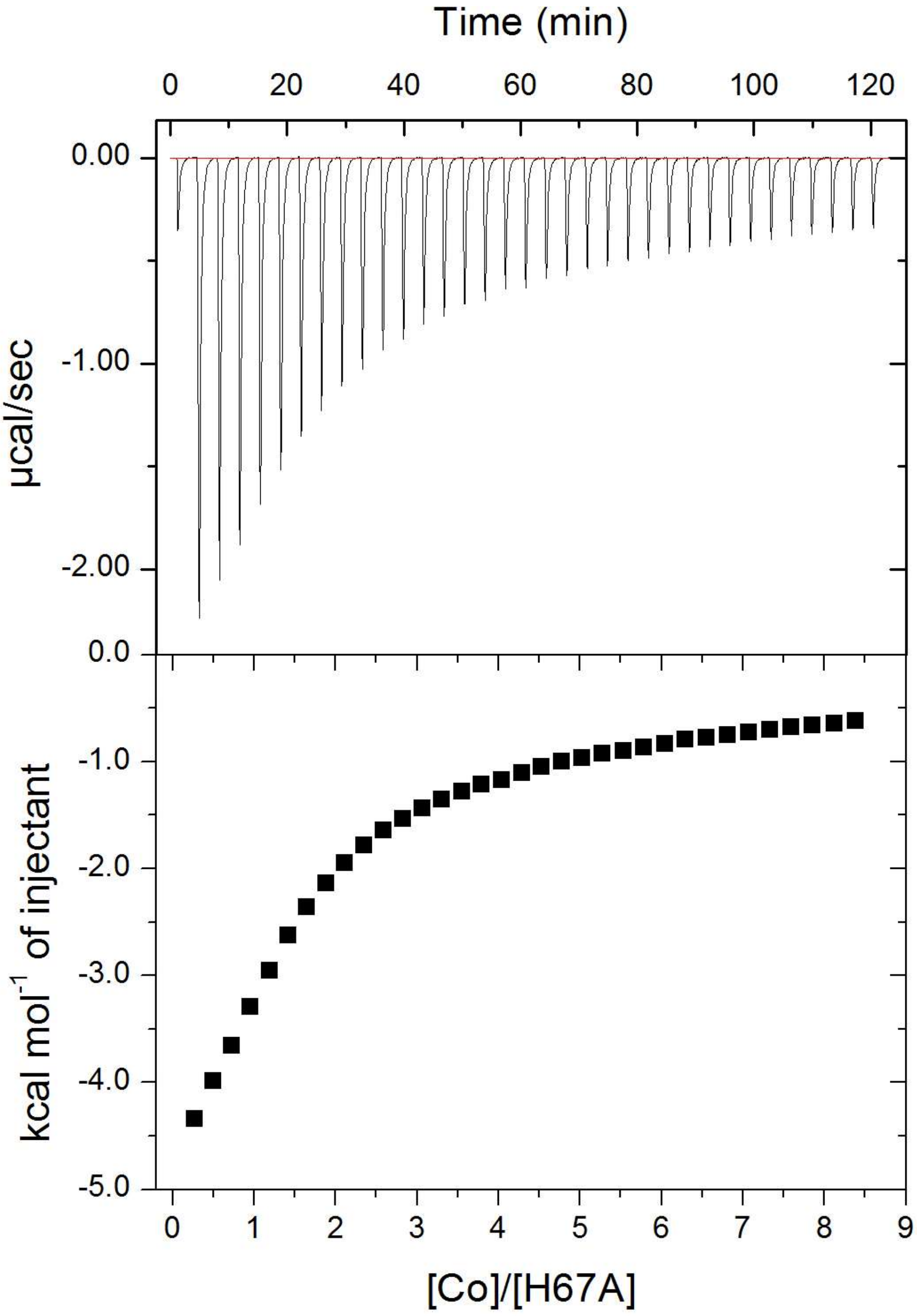

Figure S12. ITC raw data for Co^{2+} (2 mM) titration into H67A human albumin (50 µM). Upper plots: thermograms (thermal power required to maintain a zero-temperature difference between reference and sample cells in the calorimeter) showing the characteristic sequence of peaks corresponding to each ligand injection. Of note, the first titration was removed. Lower plots: binding isotherm showing normalized heats per peak as a function of molar ratio (ligand/protein) in the sample cell. Of note, the first titration point was removed for this plot. Corresponding data are shown in Figure 6 and 7C.

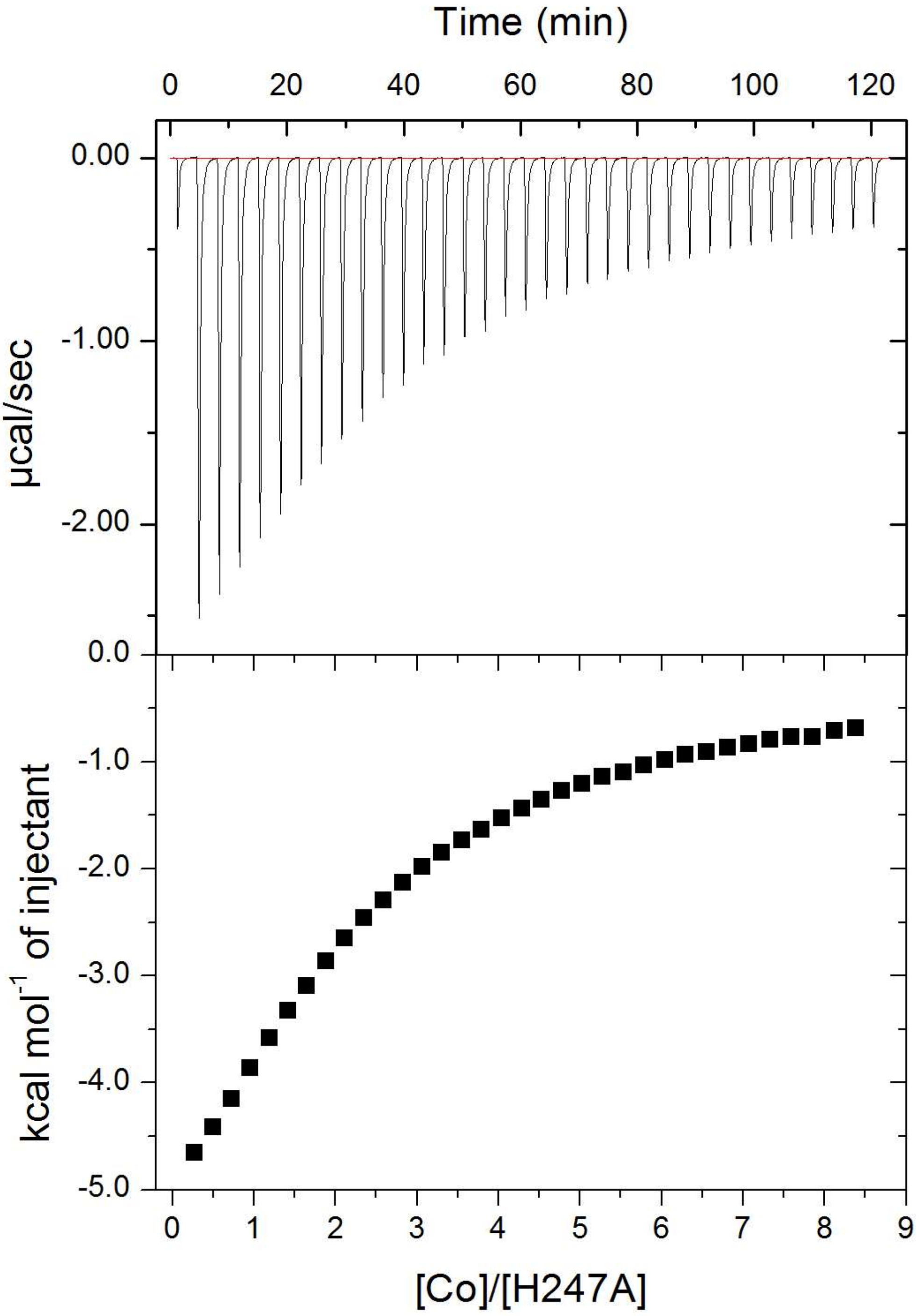

Figure S13. ITC raw data for Co^{2+} (2 mM) titration into H247A human albumin (50 µM). Upper plots: thermograms (thermal power required to maintain a zero-temperature difference between reference and sample cells in the calorimeter) showing the characteristic sequence of peaks corresponding to each ligand injection. Of note, the first titration was removed. Lower plots: binding isotherm showing normalized heats per peak as a function of molar ratio (ligand/protein) in the sample cell. Of note, the first titration point was removed for this plot. Corresponding data are shown in Figure 6 and 7D.

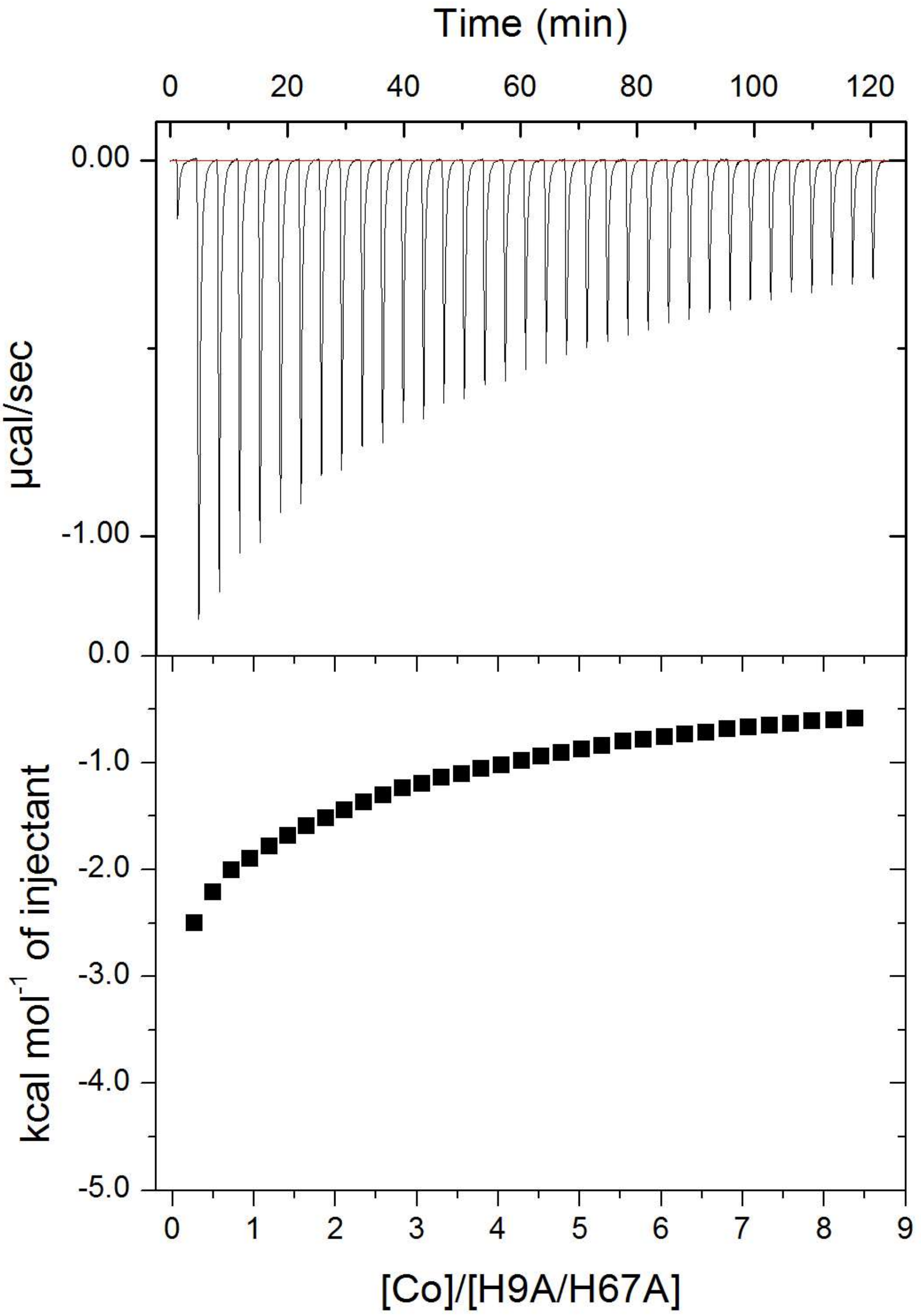

Figure S14. ITC raw data for Co^{2+} (2 mM) titration into H9A/H67A human albumin (50 µM). Upper plots: thermograms (thermal power required to maintain a zero-temperature difference between reference and sample cells in the calorimeter) showing the characteristic sequence of peaks corresponding to each ligand injection. Of note, the first titration was removed. Lower plots: binding isotherm showing normalized heats per peak as a function of molar ratio (ligand/protein) in the sample cell. Of note, the first titration point was removed for this plot. Corresponding data are shown in Figure 6.

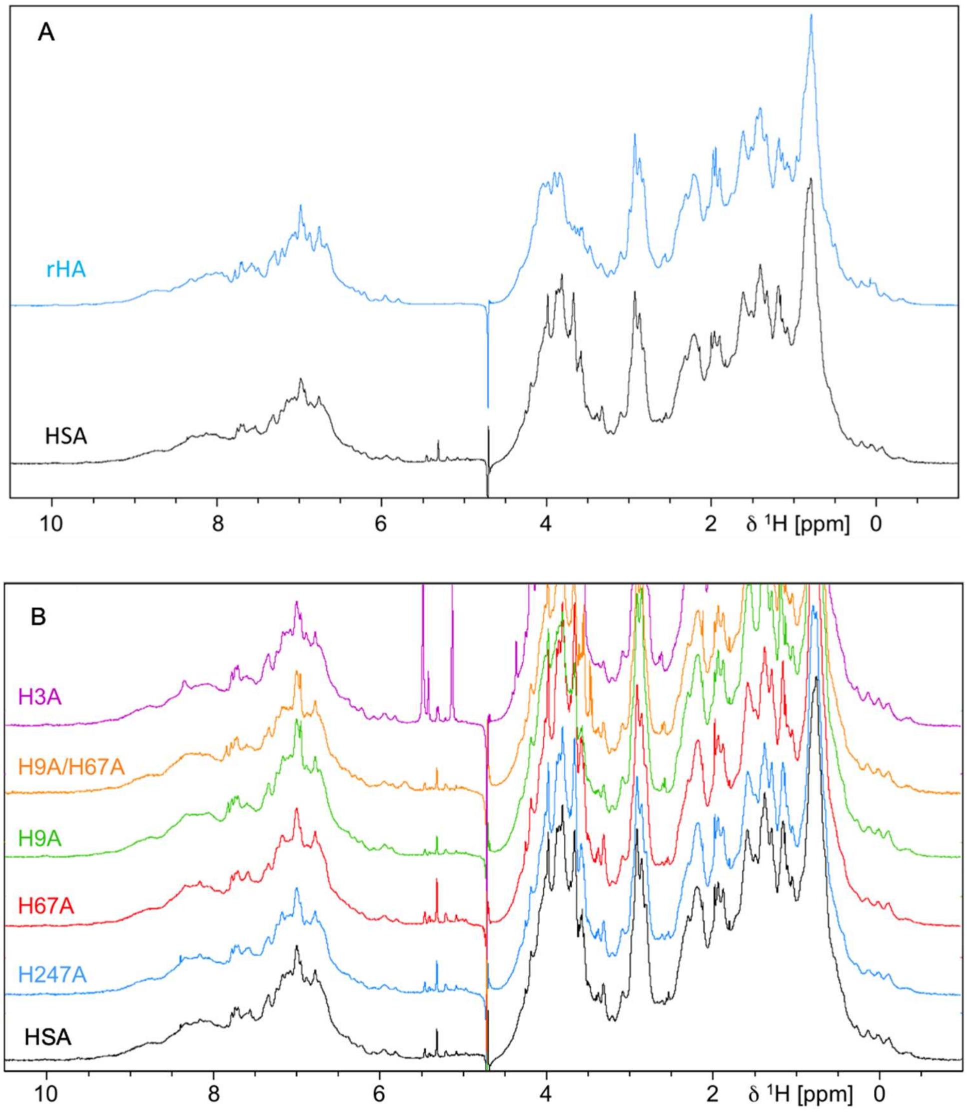

Figure S15. Assessment of protein structure in plasma-purified HSA and recombinant human albumins by ^{1}H-NMR (700 MHz). A) Comparison of the ^{1}H-NMR spectra from commercially plasma-purified HSA (HSA) and recombinant wild-type HSA (rHA). B) Comparison of ^{1}H-NMR spectra from HSA and recombinant mutant forms of HSA. The high similarity of the resonances between HSA and recombinant forms of HSA suggest that the overall fold of the proteins is similar. Of note, 0-4 ppm corresponds to the aromatic region and 6-9 ppm corresponds to the aliphatic region.

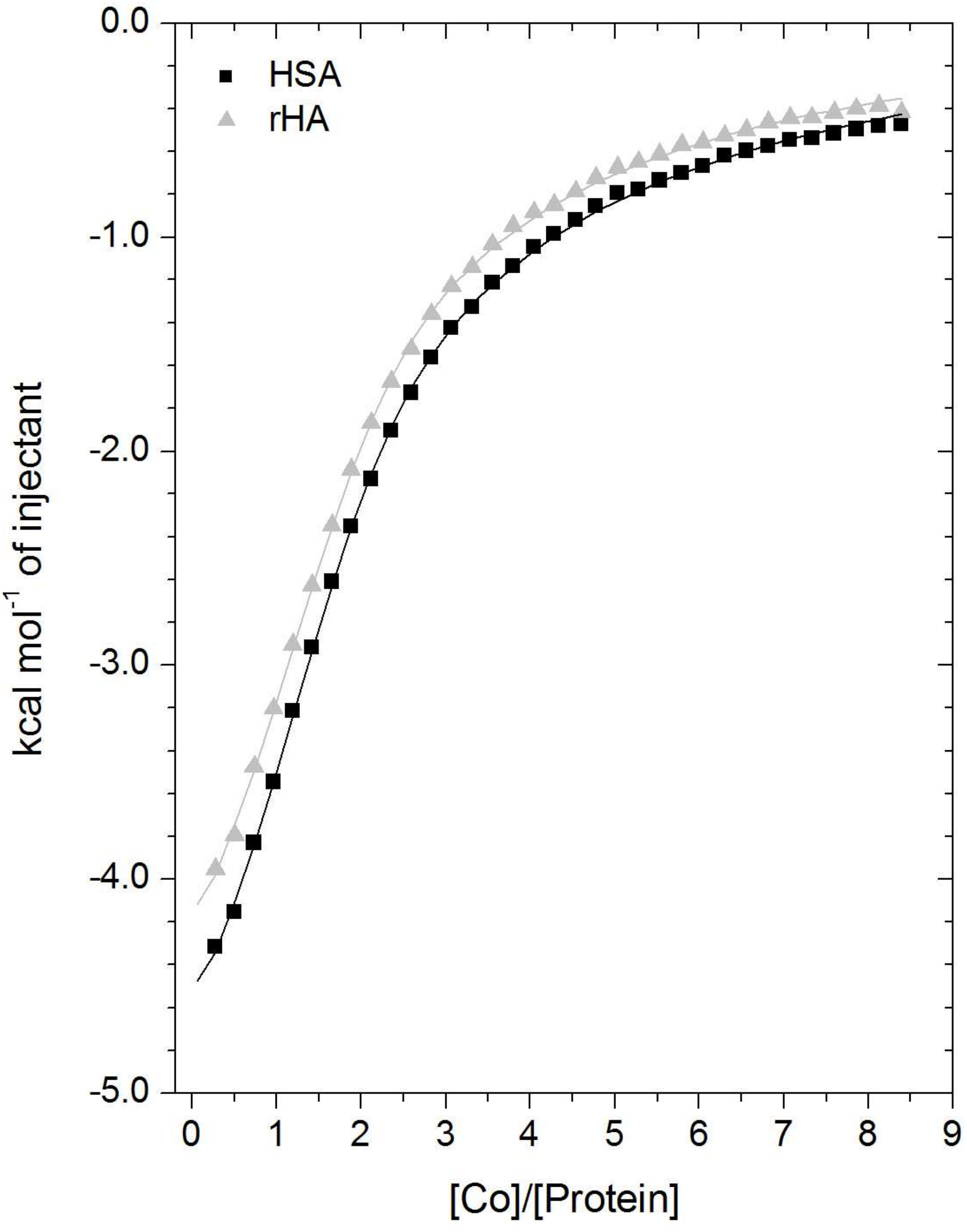

Figure S16. Measurement of Co^{2+} binding to plasma-purified HSA and recombinant wild-type human albumin (rHA) using ITC. In all cases, 50 μM of respective protein was titrated with 2 mM cobalt chloride solution for 35 injections. The data were fitted with a 3-sequential site model using MicroCal Origin 7.0 software.

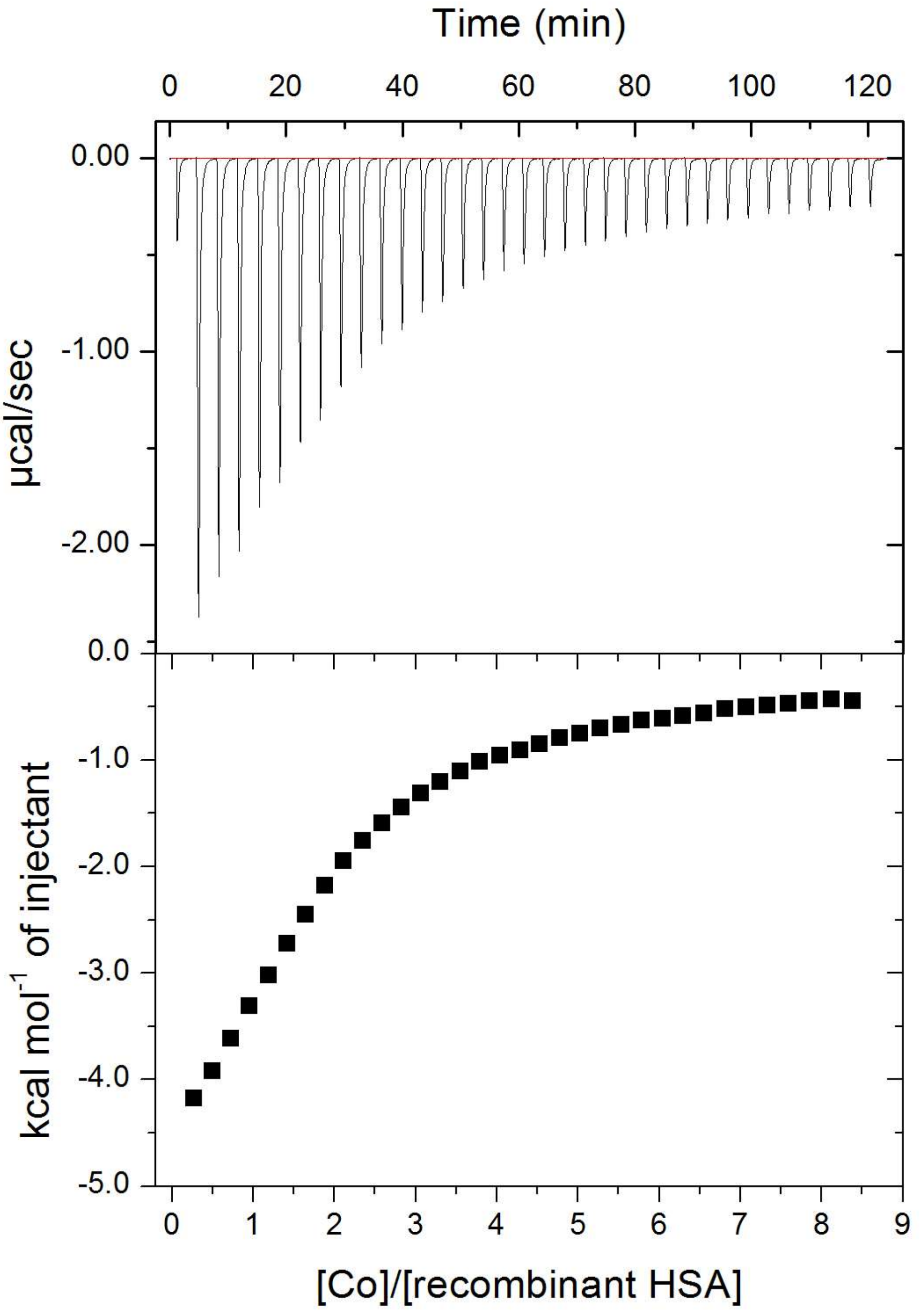

Figure S17. ITC raw data for Co^{2+} (2 mM) titration into rHA (50 µM). Upper plots: thermograms (thermal power required to maintain a zero-temperature difference between reference and sample cells in the calorimeter) showing the characteristic sequence of peaks corresponding to each ligand injection. Of note, the first titration was removed. Lower plots: binding isotherm showing normalized heats per peak as a function of molar ratio (ligand/protein) in the sample cell. Of note, the first titration point was removed for this plot.

Supplementary analysis of the circular dichroism data

Figure S18 shows the spectra obtained by titrating Co^{2+} with different HSA proteins. As previously published by Sokołowska et al. (doi: 10.1016/j.jinorgbio.2009.04.011), we observed three peaks (two negative peaks and one positive peak) with absolute $\Delta\theta$ values that increased with the addition of Co^{2+}. At first glance, it is unclear if the removal of site A, B or both induces a change in shape or intensity compared to HSA. The values for the three extrema were plotted in dependence of [Co^{2+}], and the obtained data fitted to a Hill's model to extract a Hill constant K which gave some information about the affinity of HSA for Co^{2+} ($a+b*x^n/(K^n+x^n)$). However, each binding site is expected to have a specific signature spectrum. So, measuring the affinity at each peak and comparing all four HSA proteins, might provide some insight into how much of each peak reports on which binding site. The fitting parameters are, for some data sets (H9A/H67A mainly), associated with large standard errors, this is mainly due the fact that, at 10 molar-equivalent, no plateau was reached. The K obtained for HSA obtained at each peak are very similar at about 2 mM, which is in broad agreement with the average of the three K_{ITC} obtained with the same protein (1.3 mM). Overall, the trend followed by the K values is as we would expect: $K_{H9A/H67A} > K_{H9A} > K_{H67A} > K_{HSA}$. This is assuming that site B (H9A) has higher affinity for Co^{2+} than site A (H67A) and at all three wavelengths (Table S4). Although it seems that binding at site A does not change the spectra enough at peak 1 to show a significantly lower affinity.

We further processed the data to show more precisely the specific effect of the mutations on the spectra. Figure S19 shows the result of the subtraction of the spectra of the mutated proteins from the spectrum obtained with native HSA. Between 450 nm and 800 nm some changes are observable, but no peak is clearly identifiable, and the result of the subtraction is around 0 ± 1, this indicates that binding of Co^{2+} to site A has little effect on the spectra between 450 nm and 800 nm. For H67A and the H9A/H67A double mutant a positive peak is observed at about 400 nm which is a component of peak 2. Furthermore, the H9A spectra can be separated into two groups (from 380 to 650 nm), one at lower concentrations of Co^{2+} (from 1 to 5 molar equivalent) and one at higher concentrations (from 6 to 10 molar equivalent). The first group shows little variations around 0, whereas the second group has peaks at 420 nm and 550 nm – i.e., around the same extrema observed with the non-subtracted spectra although in the reverse direction. This shows that, at these wavelengths, and specifically at higher Co^{2+} concentrations multiple binding sites are engaged with site B and having an opposite effect on the resulting spectra where all binding sites are engaged. Peak 1 (c. 325 nm) reports on both site A and B binding as well as other binding sites. The data also shows that binding at site A effect on the spectra is "stronger" than site B as H9A/H67A spectra is closer in shape to the H67A single mutant. Taken together this data shows that binding to site A or site B have different consequences on the CD spectra, which might be the result of Co^{2+} being differentially engaged with site A compared to site B.

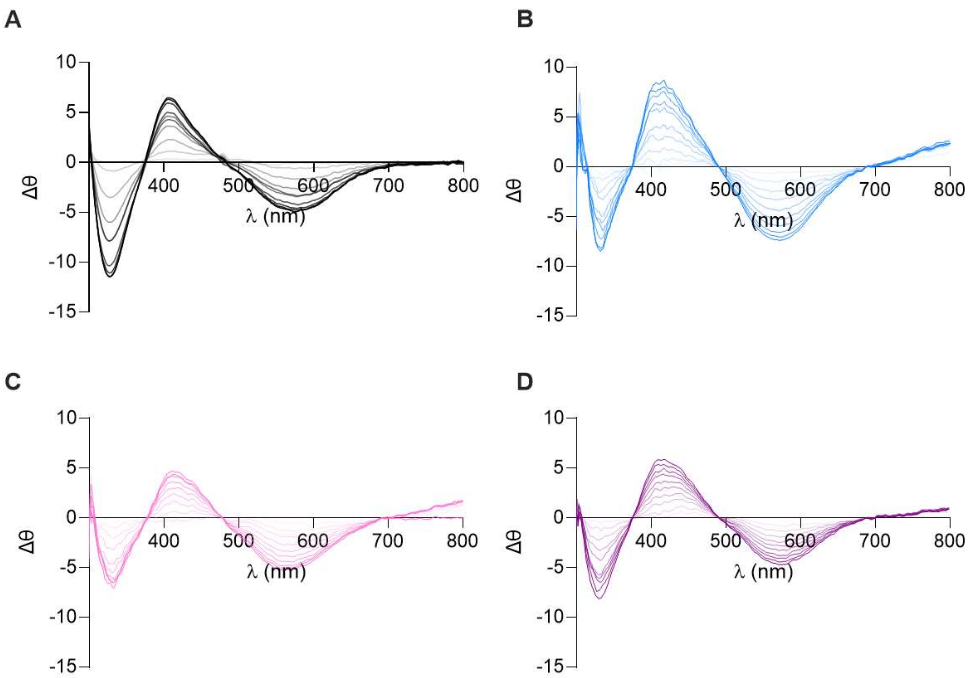

Figure S18. Baseline corrected circular dichroism spectra obtained by titration of Co^{2+} with different HSAs ([HSA] 0.5 mM, [Co^{2+}] 0.5 mM to 5 mM). (A) plasma-purified HSA, (B) H9A, (C) H67A and (D) H9A/H67A. From 0 to 10 molar equivalent, the darker the color the higher the cobalt concentration.

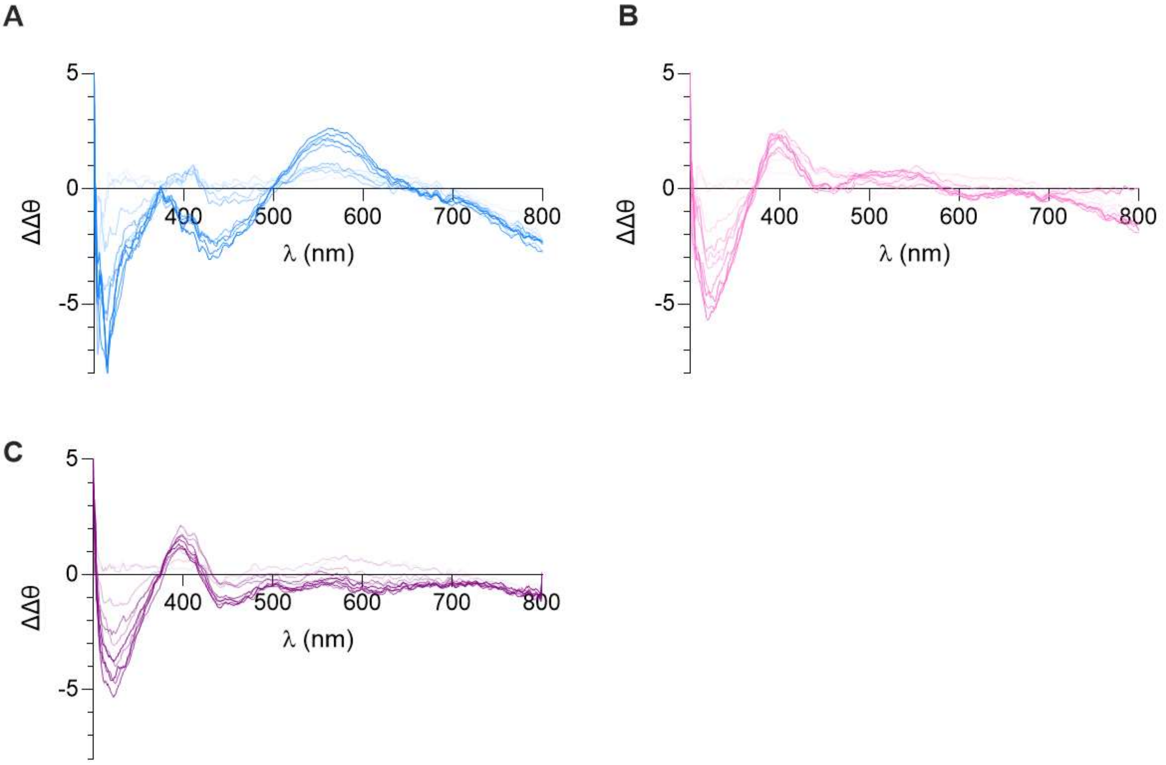

Figure S19. CD spectra obtained by subtraction of the (A) H9A, (B) H67A and (C) H9A/H67A spectra from the spectrum of HSA. Spectra are from Figure S16.

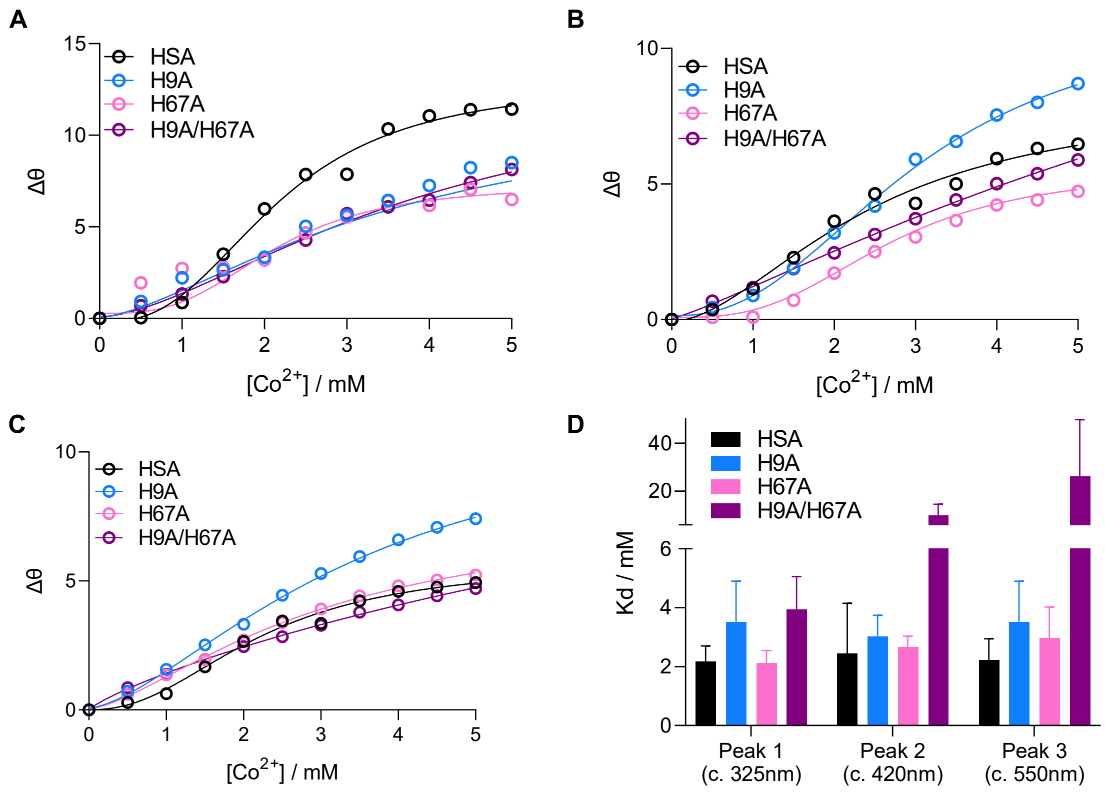

Figure S20. Plotted maxima for the three observed peaks from CD data at: (A) 325 nm, (B) 420 nm and (C) 550 nm. The modulus of the data was taken for negative peaks (325 and 550nm). The data were fitted to a Hill's model ($a+b*x^n/(Kd^n+x^n)$) using Origin 2021 software and the Kd obtained were plotted in panel D, with error bars representing the standard error. Note that Δθ represents the relative change in molar ellipticity.

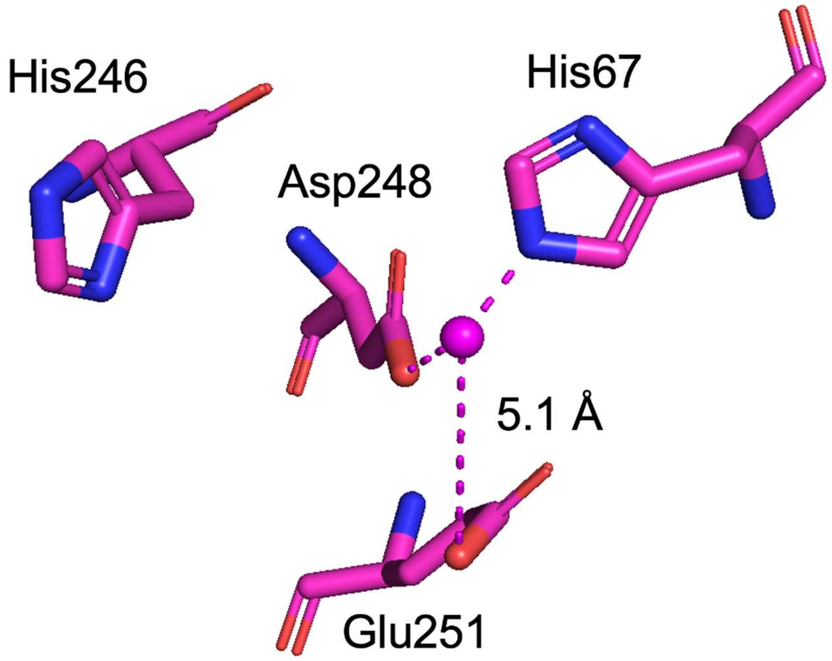

Figure S21. Co^{2+} binding at site A of 7MBL. His246 (His247 in HSA) does not participate in Co^{2+} binding at site A and loss of His246 seems to be compensated by Glu251, which is located 5.1 Å away. Residues are shown by sticks, with C in magenta, N in blue and O in red. Cobalt ion is shown as sphere in magenta.

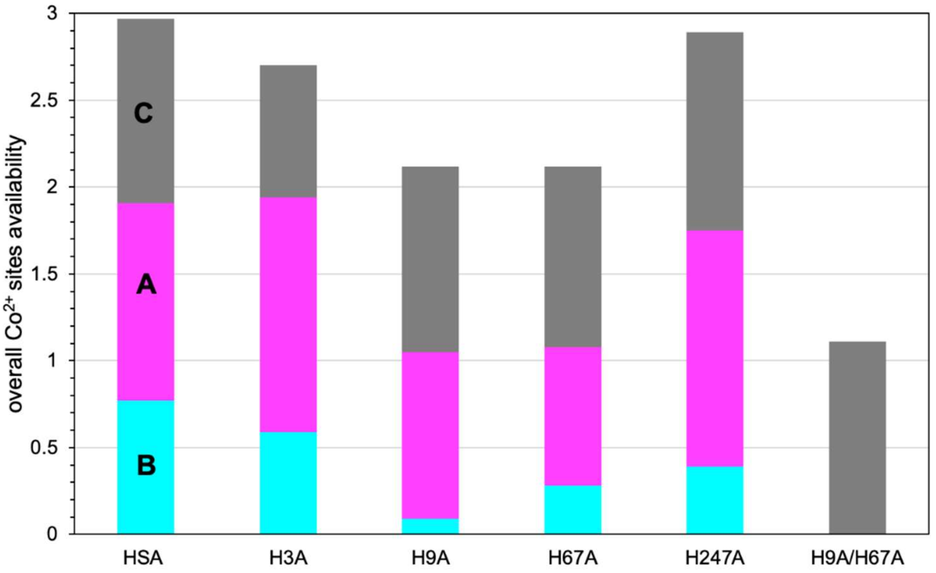

Figure S22. Overall Co^{2+}-binding capacity for HSA and mutant forms of human albumin. Availability of site A is shown as magenta; availability of site B in cyan, and availability of the weakest-affinity site(s) (labelled C) in grey.

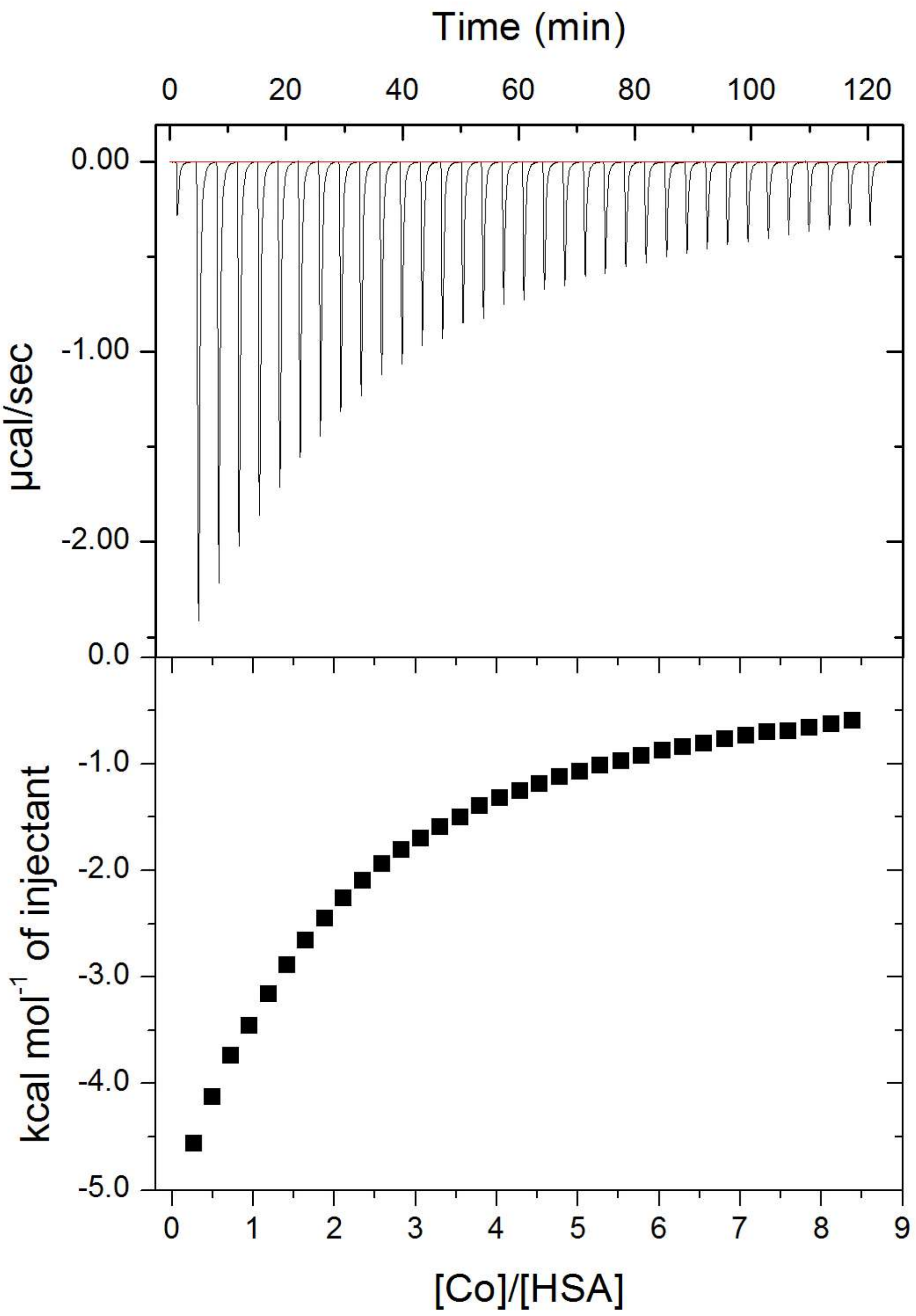

Figure S23. ITC raw data for Co^{2+} (2 mM) titration into HSA (50 µM) in presence of 5 mol. eq. of palmitate. Upper plots: thermograms (thermal power required to maintain a zero-temperature difference between reference and sample cells in the calorimeter) showing the characteristic sequence of peaks corresponding to each ligand injection. Of note, the first titration was removed. Lower plots: binding isotherm showing normalized heats per peak as a function of molar ratio (ligand/protein) in the sample cell. Of note, the first titration point was removed for this plot. Corresponding data are shown in Figure 7A.

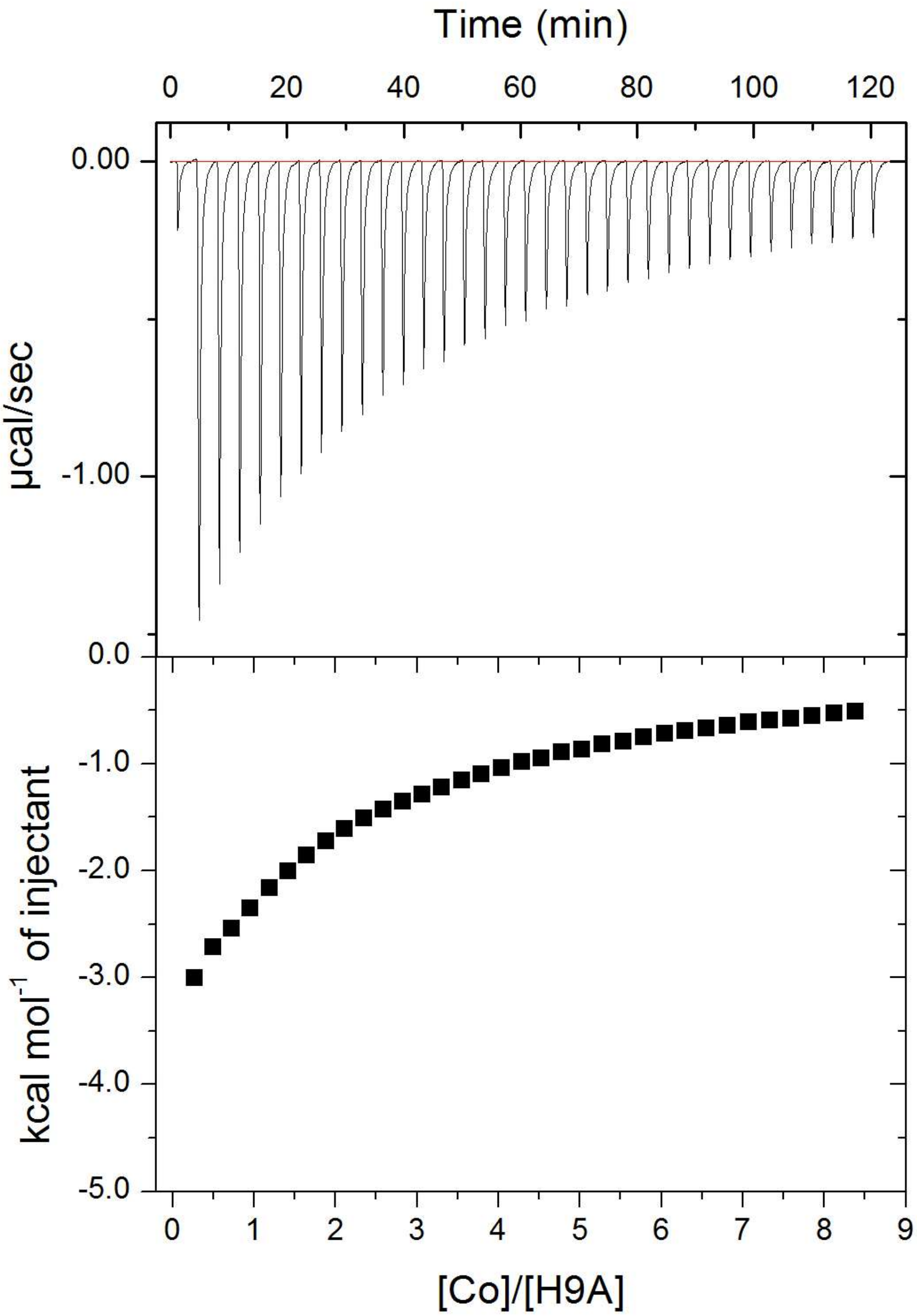

Figure S24. ITC raw data for Co^{2+} (2 mM) titration into H9A human albumin (50 µM) in presence of 5 mol. eq. of palmitate. Upper plots: thermograms (thermal power required to maintain a zero-temperature difference between reference and sample cells in the calorimeter) showing the characteristic sequence of peaks corresponding to each ligand injection. Of note, the first titration was removed. Lower plots: binding isotherm showing normalized heats per peak as a function of molar ratio (ligand/protein) in the sample cell. Of note, the first titration point was removed for this plot. Corresponding data are shown in Figure 7B.

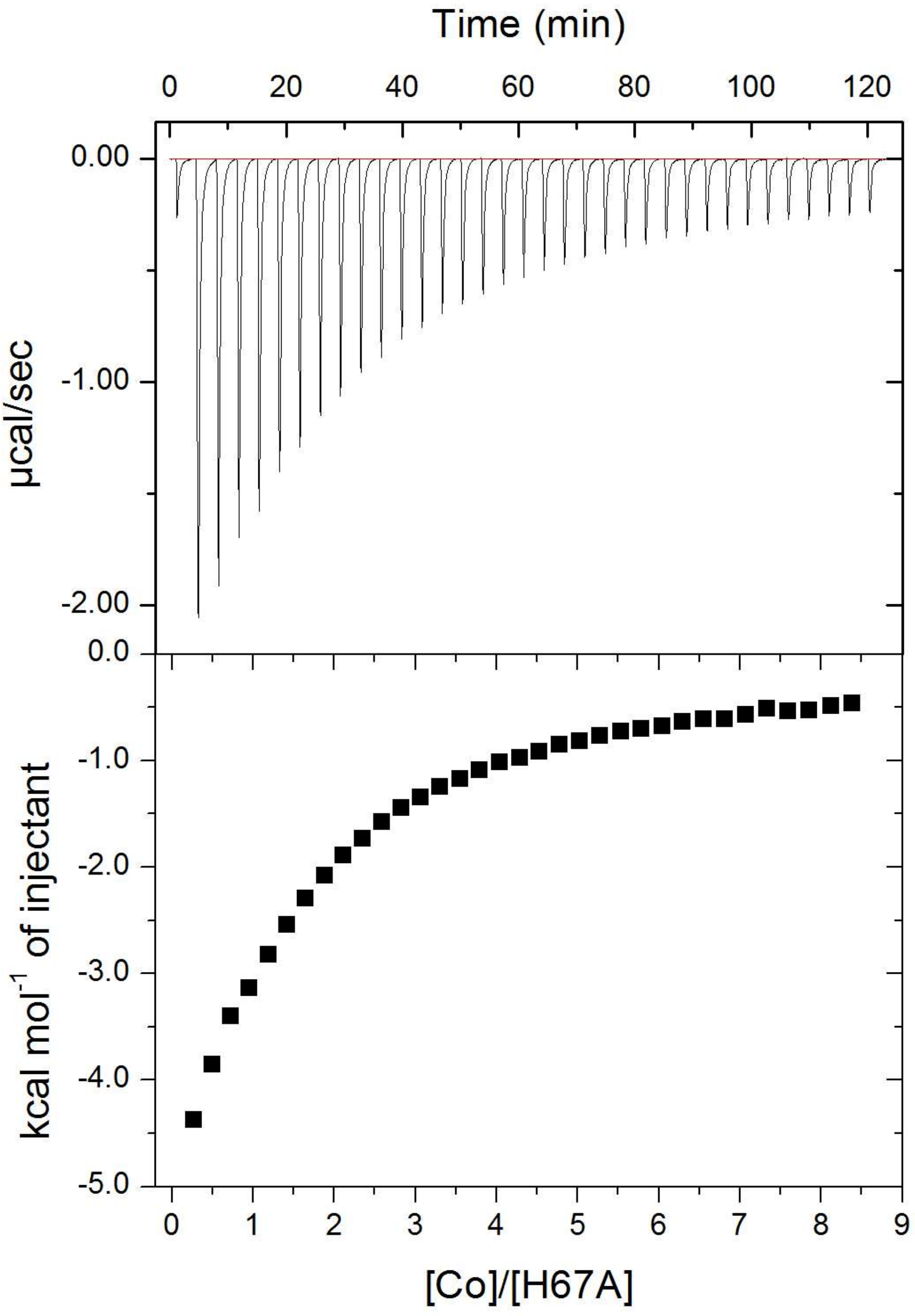

Figure S25. ITC raw data for Co^{2+} (2 mM) titration into H67A human albumin (50 µM) in presence of 5 mol. eq. of palmitate. Upper plots: thermograms (thermal power required to maintain a zero-temperature difference between reference and sample cells in the calorimeter) showing the characteristic sequence of peaks corresponding to each ligand injection. Of note, the first titration was removed. Lower plots: binding isotherm showing normalized heats per peak as a function of molar ratio (ligand/protein) in the sample cell. Of note, the first titration point was removed for this plot. Corresponding data are shown in Figure 7C.

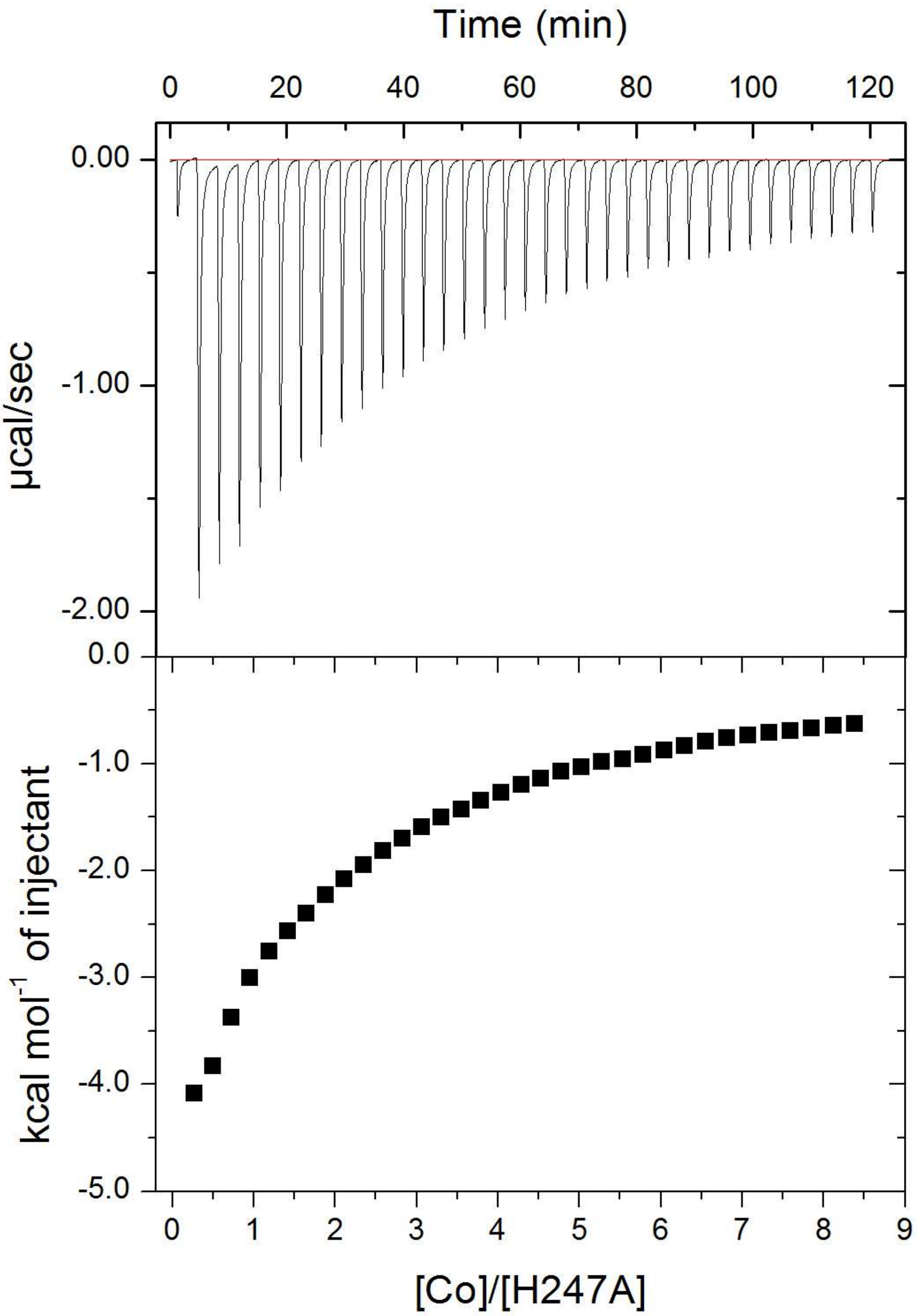

Figure S26. ITC raw data for Co^{2+} (2 mM) titration into H247A human albumin (50 µM) in presence of 5 mol. eq. of palmitate. Upper plots: thermograms (thermal power required to maintain a zero-temperature difference between reference and sample cells in the calorimeter) showing the characteristic sequence of peaks corresponding to each ligand injection. Of note, the first titration was removed. Lower plots: binding isotherm showing normalized heats per peak as a function of molar ratio (ligand/protein) in the sample cell. Of note, the first titration point was removed for this plot. Corresponding data are shown in Figure 7D.
